# Green Extraction of Bioactives from *Curcuma longa* Using Natural Deep Eutectic Solvents: Unlocking Antioxidative, Antimicrobial, Antidiabetic, and Skin Depigmentation Potentials

**DOI:** 10.3390/plants14020163

**Published:** 2025-01-08

**Authors:** Jelena Jovanović, Marko Jović, Jelena Trifković, Katarina Smiljanić, Uroš Gašić, Maja Krstić Ristivojević, Petar Ristivojević

**Affiliations:** 1 Vinča Institute of Nuclear Sciences—National Institute of the Republic of Serbia, University of Belgrade, Mike Petrovića Alasa 12–14, 11001 Belgrade, Serbia; jelena.jovanovic@vin.bg.ac.rs; 2 Innovative Centre of the Faculty of Chemistry, Ltd., University of Belgrade-Faculty of Chemistry, Studentski Trg 12-16, 11000 Belgrade, Serbia; markojovic@chem.bg.ac.rs; 3 University of Belgrade-Faculty of Chemistry, Department of Analytical Chemistry, Studentski trg 12-16, 11000 Belgrade, Serbia; jvelicko@chem.bg.ac.rs; 4 University of Belgrade-Faculty of Chemistry, Centre of Excellence for Molecular Food Sciences and Department of Biochemistry, Studentski trg 12-16, 11000 Belgrade, Serbia; katarinas@chem.bg.ac.rs (K.S.); krstic_maja@chem.bg.ac.rs (M.K.R.); 5 University of Belgrade, Institute for Biological Research “Siniša Stanković”—National Institute of Republic of Serbia, Department of Plant Physiology, Bulevar despota Stefana 142, 11108 Belgrade, Serbia; uros.gasic@ibiss.bg.ac.rs

**Keywords:** *Curcuma longa*, NADES, green extraction, curcumin content, radical scavenging activity, skin depigmentation and antidiabetic activities

## Abstract

This study evaluates the efficiency of 20 Natural Deep Eutectic Solvents (NADES) formulations for extracting curcuminoids and other bioactive compounds from turmeric and emphasize their ability to preserve and enhance antioxidant, antimicrobial, antidiabetic, and skin depigmentation effects. The NADES formulations, prepared using choline chloride (ChCl) combined with sugars, carboxylic acids, glycerol, amino acids, urea, polyols, and betaine, were assessed for their extraction efficiency based on the total phenolic content and curcumin concentration. Fourier transform infrared spectroscopy was employed to characterize the synthesized NADES and confirm their chemical composition. Bioactivity evaluations included antioxidant assays (ABTS and DPPH), antidiabetic tests (α-amylase inhibition), antimicrobial assays, and skin depigmentation (tyrosinase inhibition). The results demonstrated that NADES significantly enhanced the extraction efficiency and bioactive properties of turmeric extracts compared to water as a conventional green solvent. NADES 18 (ChCl/1,2-propanediol/water 1:1:1) and NADES 19 (glycerol/betaine/water 1:1:3) exhibited the highest extraction yields, with curcumin concentrations of 30.73 ± 1.96 mg/g and 31.70 ± 2.02 mg/g, respectively, outperforming water (26.91 ± 1.72 mg/g), while NADES 17 (ChCl/1,2-propanediol/water 0.5:3:0.5:5) and NADES 20 (glycerol/lysine/water 1:1:3) exhibited the most potent antioxidant activity. Furthermore, NADES 14 (ChCl/lactic acid/water 1:2:5) demonstrated the strongest tyrosinase inhibition (98.7%), supporting its potential for skin-brightening applications, including notable α-amylase inhibition exceeding 90%. This study aligns with the principles of green chemistry, as NADES are effective and sustainable solvents for natural product extraction. The presenting benefits of improved extraction efficiency and enhanced bioactivities position NADES as a promising and eco-friendly approach for developing efficient bioactive compound extraction methodologies.

## 1. Introduction

Turmeric (*Curcuma longa*), a member of the Zingiberaceae family, is highly valued not only as a culinary spice but also for its extensive medicinal applications, especially in traditional Indian and Chinese medicine [1]. The largest exporters of turmeric are India (over 160,000 tons annually), Myanmar (over 29,300 tons annually) and the Netherlands (over 4500 tons annually) [2]. Among the various species within the Curcuma genus, *Curcuma longa* is the most extensively studied due to its high curcuminoid content, which makes up 3–5% of the rhizome. Curcumin, the principal bioactive compound in turmeric, comprises about 75% of these curcuminoids. Chemically known as diferuloylmethane, curcumin is a polyphenol widely recognized for its diverse pharmacological effects, including strong antioxidant, antimicrobial, anti-inflammatory, and anticancer properties [3]. Turmeric has long been utilized in traditional medicine for managing bronchial asthma, enhancing liver function, treating depressive disorders, cardiovascular diseases, and arthritis, reducing the risk of heart disease and addressing various other health conditions. These beneficial properties have brought curcumin, its active compound, into focus for potential use in the food, cosmetic, and pharmaceutical sectors [4].

Conventional extraction methods, such as solid–liquid extraction, sonication, Soxhlet extraction, and similar techniques, often rely on the use of organic solvents, including ethanol, methanol, acetone, and hexane, as well as water. While widely utilized for the extraction of curcumin from *C. longa*, these solvents possess several limitations, including lengthy extraction times, low selectivity, and the potential degradation of heat-sensitive compounds [5,6]. Advanced extraction technologies such as ultrasound-assisted extraction, microwave-assisted extraction, enzyme-assisted extraction, pressurized liquid extraction and supercritical fluid extraction have been developed to address these challenges [7]. These novel methods demonstrate enhanced efficiency and selectivity, achieving greater accuracy, higher yields, and significantly reduced extraction times compared to traditional techniques, while aligning with the growing emphasis on eco-friendly green technologies [8]. To minimize environmental contamination, “green solvents” have emerged as a preferred alternative to traditional organic solvents, offering a more sustainable and eco-friendly solution. Natural Deep Eutectic Solvents (NADES) have gained attention as promising alternatives to traditional organic solvents for the extraction of bioactives, including curcumin. NADES are composed of naturally occurring, non-toxic substances like sugars, organic acids, amino acids, and choline derivatives. They provide significant benefits, including low toxicity, biodegradability, and enhanced extraction efficiency, positioning them as an eco-friendly and effective option [9]. NADESs, especially those based on organic acids, exhibit strong antioxidant activity, enhancing food stability and shelf life. They also show significant antibacterial properties against Gram-negative bacteria by interacting with and damaging bacterial cell walls [10,11]. The use of NADES in curcumin extraction is particularly noteworthy as it adheres to the principles of green chemistry, providing a more sustainable and environmentally friendly method for obtaining high-purity curcumin for a wide range of industrial applications [12]. The unique physicochemical properties of NADES derive from the formation of eutectic mixtures through interactions between hydrogen bond donors (HBDs) and hydrogen bond acceptors (HBAs). This process results in solvents with melting points significantly lower than their components [13]. This characteristic enables NADES to function efficiently at lower temperatures, preserving the stability of heat-sensitive compounds such as curcumin [14]. Recent studies have demonstrated that NADES are highly effective for extracting curcumin, achieving superior extraction efficiencies and better preservation of curcumin’s bioactive properties compared to traditional solvent systems [15]. These findings underscore the potential of NADES as a green and efficient solvent system for curcumin extraction, contributing to the development of more sustainable extraction processes in various industries [16,17,18].

To the best of our knowledge, no studies have explored the antimicrobial, antioxidative, depigmentation, and antidiabetic activities of turmeric bioactives extracted with NADES. We propose that NADES may significantly improve the extraction efficiency and stability of curcumin from turmeric compared to traditional solvents, thus maintaining its powerful biological activities, including radical scavenging potential antimicrobial, depigmentation and antidiabetic activities. These solvents may also contribute to improved bioavailability and efficacy of bioactives in various therapeutic and functional food applications. This improvement is expected to preserve or even enhance bioactive antioxidant properties, providing a sustainable and eco-friendly alternative for the extraction of bioactive compounds. The study aims to investigate the effectiveness of 20 different NADES formulations in extracting curcumin from turmeric, evaluating each solvent’s impact on turmeric’s antioxidant activity. The goal is to identify optimal NADES formulations that maximize extraction efficiency and bioactive preservation, contributing to developing greener, more sustainable extraction methods for bioactive compounds in alignment with green chemistry principles. Its enhanced stability and bioactivity make it suitable for therapeutic formulations, dietary supplements, and natural colorants in food products.

## 2. Results and Discussion

### 2.1. Characterizations of NADES

Various NADES were prepared using combinations of choline chloride (ChCl), polyhydroxy alcohols (glycerol, xylitol, and 1,2-propanediol), sugars (fructose, and glucose), carboxyl acids (citric acid, and lactic acid), amino acids (betaine, and lysine), urea, and water, utilizing different molar ratios of hydrogen bond acceptors (HBAs) and hydrogen bond donors (HBDs). The study focused on evaluating their effectiveness as extraction agents for bioactive compounds. Fourier transform infrared (FTIR) spectroscopy was employed to characterize the synthesized NADES and confirm their chemical composition. The infra-red spectra in Figure S1 (Supplementary Materials), show the characteristic peaks of each NADES component, while Figure S2 (Supplementary Materials) shows the spectra of the NADES mixtures. The FTIR spectra exhibited characteristic peaks corresponding to the functional groups of the NADES constituents. Broad -OH stretching bands (3200–3500 cm^−1^) confirmed the presence of hydroxyl groups from polyols (glycerol, fructose, glucose) and water, indicative of extensive hydrogen bonding networks within the NADES. A bathochromic shift of the -OH stretching band was observed in ChCl-containing NADES, suggesting hydrogen bond formation. Strong C=O stretching bands (1700–1730 cm^−1^) were observed in NADES containing carboxylic acids (lactic acid, citric acid). C-O stretching vibrations (1000–1200 cm^−1^) were attributed to glycerol, urea, and 1,2-propanediol. N-H stretching (3200–3500 cm^−1^) and bending (1550–1650 cm^−1^) vibrations were observed in NADES containing amines or amides (lysine, urea). Characteristic peaks associated with the zwitterionic structure of betaine were observed, including asymmetric -COO^−^ stretching (1600–1650 cm^−1^) and symmetric stretching (1400–1450 cm^−1^).

For NADES 8 (ChCl/glycerol 1:1), NADES 9 (ChCl/glycerol 1:2) and NADES 10 (ChCl/glycerol 1:3) (Figure S2e–g), the broad O-H stretching band (~3200–3600 cm^−1^) intensified with increasing glycerol content, indicating the formation of glycerol-dominated hydrogen bonds. In parallel, the C-O stretching band (~1000–1300 cm^−1^) also showed enhanced intensity, emphasizing the role of the hydroxyl groups of glycerol, while minimal shifts in the C-N stretching region (~900–1100 cm^−1^) highlighted minimal variations in the ChCl–glycerol interaction [19]. The FTIR spectra (Figure S2a–c) of systems involving ChCl, glycerol, and water (1:1:5, 1:2:5, and 1:1:2), i.e., NADES 1 (ChCl/glycerol/water 1:1:5), NADES 3 (1:2:5) and NADES 5 (1:1:2), indicate that different proportions of glycerol and water affect hydrogen bonding and molecular interactions [20]. In the 1:1:5 mixture, the spectrum shows prominent contributions from water, with the O-H band appearing more intense, indicating extensive hydrogen bonding mediated by water. The C-O stretching band (~1000–1300 cm^−1^) and the C-N stretching band (~900–1100 cm^−1^) remain relatively consistent. At the 1:2:5 ratio, increasing glycerol content enhances the self-association of glycerol molecules, as seen in a very slight shift of the O-H band to lower wavenumbers. At the 1:1:2 ratio, ChCl–glycerol interactions dominate, with a reduced contribution from free water. The spectral changes at these ratios illustrate the role of water in modulating hydrogen bonding, while glycerol also increases structural complexity at higher concentrations [21]. Similar trends were observed in spectra (Figure S2h–j) for ChCl-based NADES with fructose, glucose, or xylitol: NADES 2 (ChCl/fructose/water 1:2:5), NADES 4 (ChCl/glucose/water 1:1:5) and NADES 12 (ChCl/xylitol/water 1:1:5). In the fructose system, changes in the C-O-C and C=O stretching regions (1050–1150 cm^−1^) suggest disruption of the cyclic fructose structure and formation of new hydrogen bonds, while the glucose-based NADES showed prominent shifts in the ~1000–1100 cm^−1^ region, reflecting interactions of hydroxyl and aldehyde groups [22]. Xylitol, with its linear structure, showed simpler spectral patterns, with dominant peaks corresponding to -OH and C-O stretching vibrations, forming fewer hydrogen bonds compared to cyclic sugars [23].

Systems involving ChCl with citric acid, urea, lactic acid or 1,2-propanediol also showed unique spectral features. For example, NADES with ChCl, citric acid and water, such as NADES 6 (ChCl/citric acid/water 1:2:5) and NADES 7 (ChCl/citric acid/water 2:1:5) showed significant contributions to O-H and C=O stretching due to the hydroxyl and carboxyl groups of citric acid (Figure S2l,m), an identical trend was observed in the system with lactic acid, i.e., NADES 14 (ChCl/lactic acid/water 1:2:5), where an upward shift in the carboxyl group stretching vibration band of ~1750 cm^−1^ was observed, along with a similar change in the C-N stretching band of ~1400 cm^−1^ (Figure S2k). In addition, a downward shift in the hydroxyl group vibration band was observed. These spectral changes confirm the formation of hydrogen bonds between ChCl and lactic acid, providing evidence for the interaction between the components. The obtained results are consistent with previously reported data [24]. In systems with urea or lysine (Figure S2q–t), i.e., NADES 11 (glycerol/urea/water 1:1:2), NADES 16 (ChCl/urea/water 1:2:5) and NADES 20 (glycerol/lysine/water 1:1:3), noticeable shifts in the N-H and C=O stretching regions highlighted the influence of amide or zwitterionic groups in hydrogen bond formation [25]. Together, these spectra illustrate how different compositions modulate hydrogen bond networks, providing insight into the modified properties and applications of NADES.

### 2.2. Antioxidant Potential of Turmeric NADES Extracts

#### 2.2.1. Total Phenolic Content (TPC) of Extracts

The green NADES extracts were analysed using spectrophotometric assays to assess their bioactive composition, with a particular emphasis on TPC as a main contributor to potential health benefits [26]. The NADES turmeric extracts exhibited TPC values ranging from 6.92 to 54.77 mg GAE/g (Table 1). Comparing the extraction efficiency of twenty NADES formulations and water as a reference solvent, NADES 2 (ChCl/fructose/water 1:1:5) and NADES 20 (glycerol/lysine/water 1:1:3) shows significantly higher TPC values compared to water, making them more efficient extraction media than conventional solvent. Water was selected as the control solvent due to its recognition as a conventional and environmentally sustainable option. However, its extraction efficiency is typically inferior to that of organic solvents. In this context, the aforementioned NADES formulations demonstrated superior performance in phenol extraction, addressing the limitations of water while offering a green and efficient alternative for the extraction of these compounds. The literature highlights the enhanced efficiency of sugar-based NADES for phytonutrient extraction [27]. The higher efficiency of the ChCl–fructose–water system compared to NADES 4 (ChCl/glucose/water 1:1:3) (6.92 ± 0.14 mg GAE/g) was attributed to its increased water content, which reduces viscosity and enhances mass transfer while maintaining the eutectic structure [28].

NADES 20 (glycerol/lysine/water 1:1:3) demonstrated the second-highest phenolic extraction efficiency (36.84 ± 0.13 mg GAE/g). Glycerol’s polarity and strong hydrogen-bonding capacity solubilize phenolics, while lysine’s basicity enhances interactions with acidic phenolic compounds, improving yields. NADES systems incorporating lysine show promising properties for antioxidant extraction [14]. In contrast, systems composed of ChCl and glycerol, i.e., NADES 8 (ChCl/glycerol 1:1), NADES 9 (ChCl/glycerol 1:2) and NADES 10 (ChCl/glycerol 1:3) demonstrated lower phenolic yields (9.41 ± 0.59 mg GAE/g, 17.76 ± 0.17 mg GAE/g and 14.88 ± 0.07 mg GAE/g, respectively), but increased glycerol content improved extraction efficiency due to reduced viscosity [29]. Similarly, adding water to the choline chloride–glycerol mixtures decreased viscosity and increased TPC, consistent with trends observed in systems involving organic acids [28].

#### 2.2.2. Radical Scavenging Activity

Considering the high phenolic content of turmeric and its extensively documented radical scavenging activity, as demonstrated in previous studies [30,31], the antioxidant capacity of green NADES extracts was assessed through 2,2-diphenyl-1-picrylhydrazyl (DPPH) and 2,2’-azino-bis(3-ethylbenzothiazoline-6-sulfonic acid) (ABTS) radicalassays, demonstrating the efficacy of NADES in neutralizing free radicals. The results of antioxidant assays show DPPH and ABTS radical scavenging activity in the ranges of 4.88–30.50 μmol TE/g, and 0.22–14.55 μmol TE/g, respectively. NADES 17 (ChCl/glycerol/citric acid/water 0.5:2:0.5:5) (30.5 ± 1.0 μmol TE/g), NADES 20 (glycerol/lysine/water 1:1:3) (29.6 ± 0.3 μmol TE/g), NADES 11 (glycerol/urea/water 1:1:2) (24.1 ± 0.6 μmol TE/g), NADES 15 (ChCl/citric acid/water 1:1:5) (22.1 ± 0.2 μmol TE/g), and NADES 7 (ChCl/citric acid/water 2:1:5) (20.4 ± 4.5 μmol TE/g), showed equivalent DPPH activity compared to water (26.6 ± 0.3 μmol TE/g), with superior activity of NADES 17 and NADES 20 outperforming the water extract (Table 1). The highest ABTS activity was observed in NADES 11 (glycerol/urea/water 1:1:2) (4.74 ± 0.14 μmol TE/g), NADES 20 (glycerol/lysine/water 1:1:3) (4.64 ± 0.03 μmol TE/g), NADES 17 (ChCl/glycerol/citric acid/water 0.5:2:0.5:5) (4.62 ± 0.07 μmol TE/g) and NADES 19 (glycerol/betaine/water 1:1:3) (4.35 ± 0.03 μmol TE/g) (Table 1). These formulations, together with NADES 5 (ChCl/glycerol/water 1:1:2) (4.29 ± 0.02 μmol TE/g), NADES 3 (ChCl/glycerol/water 1:2:5) (4.25 ± 0.08 μmol TE/g) and NADES 4 (ChCl/glucose/water 1:1:3) (4.20 ± 0.16 μmol TE/g), demonstrated comparable antioxidant activities as water extract (Table 1). NADES 20 emerged as a highly effective solvent for extracting phenolics and antioxidants, with the literature confirming lysine-based NADES superior performance over conventional solvents and other amino acid-based NADES, such as arginine and proline [32]. These findings underscore the need to consider phenolic content and antioxidant activity across methods to evaluate extraction efficiency. Each assay targets different interactions and mechanisms, providing complementary insights [33,34].

### 2.3. Antibacterial Activity of NADES Extracts

The agar well diffusion method was employed to assess the antibacterial activity of the turmeric extracts under investigation. This method provides a clear visualization of inhibition zones, enabling qualitative comparisons of the in vitro antimicrobial potential between the extracts [35].

As shown in Table 2, the tested extracts exhibited greater antibacterial activity against *L. monocytogenes* compared to *S. typhimurium*. The higher sensitivity of Gram-positive strains is consistent with the literature reports [36,37], attributing this to the structural differences in the bacterial cell wall. Gram-positive bacteria have a thick peptidoglycan layer but lack the outer membrane present in Gram-negative bacteria. The absence of this outer membrane facilitates easier penetration of antimicrobial agents into Gram-positive cells, while the lipopolysaccharide-based outer membrane in Gram-negative bacteria acts as a barrier, impeding many compounds from reaching intracellular targets [38].

Turmeric was shown to possess significant antimicrobial activity against various pathogens, including bacteria, fungi, and viruses. This activity is attributed not only to curcumin but also to other phytochemicals present in turmeric, such as tannins, alkaloids, phenols, steroids, flavonoids, phlorotannin, cardiac glycosides, terpenoids, triterpenes, saponins [39,40].

The extracts obtained with NADES composed of ChCl and citric acid, i.e., NADES 6 (ChCl/citric acid/water 1:2:5), NADES 7 (ChCl/citric acid/water 2:1:5), NADES 13 (ChCl/citric acid/water 1:2:3), NADES 15 (ChCl/citric acid/water 1:1:5) and NADES 17 (ChCl/glycerol/citric acid/water 0.5:2:0.5:5) exhibited the highest antibacterial activity against both tested strains. In contrast, extracts obtained with NADES 11 (glycerol/urea/water 1:1:2), NADES 16 (glycerol/urea/water 1:2:5), NADES 19 (glycerol/betaine/water 1:1:3), NADES 12 (ChCl/xylitol/water 1:1:5) and NADES 18 (ChCl/1,2-propanediol/water 1:1:1) demonstrated limited activity, which was restricted to *L. monocytogenes*. All other extracts based on ChCl and glycerol, as well as the aqueous extract, showed no detectable antibacterial activity.

Additionally, NADES can contribute to antimicrobial activity. Thus, beyond the plant-derived compounds, the inhibitory zone dimensions may vary depending on the NADES components used for extraction [41]. As seen in Table 2, extracts containing citric acid as one of the NADES components displayed notable antibacterial activity against both bacterial strains. The literature highlights the strong antimicrobial effects of organic acid-based NADES, which may be explained by their low pH (<3), increasing their toxicity toward bacterial cells, whose optimal pH range for growth is 6.5–7.5. This phenomenon is supported by De Morais et al., who demonstrated that organic acids in NADES cause protein denaturation and reduce bacterial cell activity [42]. Furthermore, NADES 4 (ChCl/glucose/water 1:1:3), containing 20% glucose, exhibited pronounced inhibition against *S. typhimurium*, likely due to an osmotic pressure effect. Cvjetko Bubalo et al. reported that carbohydrate-based NADES induce cell dehydration and lysis by generating high osmotic pressure [43]. However, the lack of antibacterial activity in NADES 2 (ChCl/fructose/water 1:1:5) may be due to a reduced proportion of the sugar component compared to glucose-based NADES (14%).

It is important to highlight that both *S. typhimurium* and *L. monocytogenes* are significant foodborne pathogens. *S. typhimurium* primarily affects the intestines but can also cause more severe systemic infections, whereas *L. monocytogenes* is more likely to lead to severe invasive infections, particularly in vulnerable populations such as the elderly, pregnant women, and immunocompromised individuals [44]. Considering these factors, the use of NADES extracts in the food industry presents a promising alternative to conventional artificial preservatives, potentially enhancing food safety by targeting these pathogens.

### 2.4. Inhibitory Effects of NADES Extracts on Tyrosinase Activity and Keratinocyte Survival

Tyrosinase is a key enzyme regulating the rate-limiting step in melanin biosynthesis, making it a crucial biological target for the development of skin-whitening and anti-melanogenic agents [45]. Rocchitta et al. confirmed the anti-tyrosinase activity of turmeric metabolites, particularly highlighting curcumin, where the β-diketone moiety of its aliphatic chain plays an essential role in enzyme inhibition [46]. Athipornchai et al. also investigated phenolic analogues of curcumin, demonstrating that these compounds have IC_50_ values up to 20 times higher than the reference standard, kojic acid [47]. Given these properties, NADES extracts of turmeric may offer enhanced tyrosinase inhibitory effects. In addition to enabling efficient extraction of active compounds, the bioactivity of NADES components themselves could synergistically enhance the efficacy of these extracts in skin applications and antioxidant functions.

**Figure 1:**
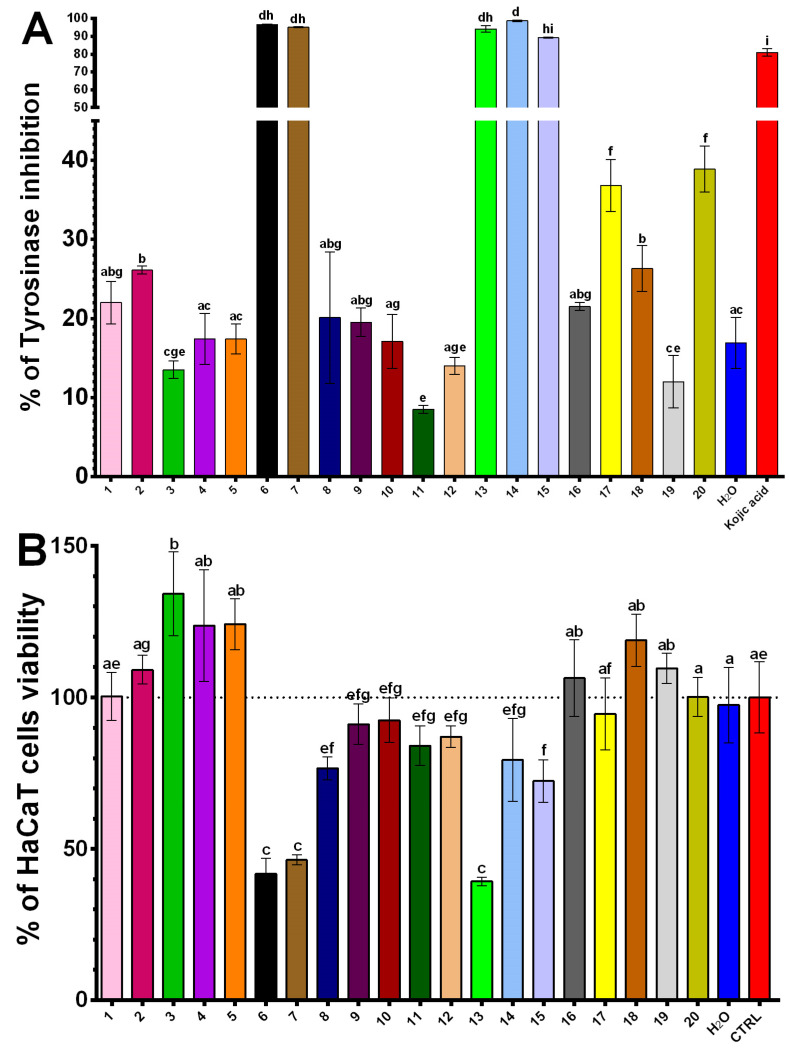
Tyrosinase enzyme test inhibition and HaCaT cells’ survival under the action of 20 NADES and conventional turmeric extracts. (**A**) percentage of tyrosinase inhibition with Kojic acid as reference standard. (**B**) Cytotoxicity test reflecting survival of spontaneously immortalized, human keratinocyte cell line (HaCaT). Distinct letters above the columns indicate statistically significant differences (*p* < 0.05) as determined by Tukey’s multiple comparisons test. The dashed line marks 100% viability of untreated control cells.

The results in Figure 1 indicate significant inhibitory activity of NADES turmeric extracts against tyrosinase. Extracts were obtained with NADES 6 (ChCl/citric acid/water 1:2:5), NADES 7 (ChCl/citric acid/water 2:1:5), NADES 13 (ChCl/citric acid/water 1:2:3), NADES 14 (ChCl/lactic acid/water 1:2:5) and NADES 15 (ChCl/citric acid/water 1:1::5) exhibited strong inhibition, with values of 96.7%, 95.1%, 94.2%, 98.7%, and 89.2%, respectively (Table S1). Notably, NADES 6, 7, 13 and 15 contained citric acid, a widely recognized anti-pigmentation agent in the cosmetic industry [48], while NADES 14 contained lactic acid, whose inhibitory activity has also been documented [49]. These extracts demonstrated superior inhibition compared to kojic acid. In contrast, water extract exhibited significantly weaker tyrosinase inhibition.

The evaluation of tyrosinase inhibition, including its role in skin whitening, must be considered alongside cytotoxicity testing on skin cells, particularly keratinocytes. Accordingly, the cytotoxicity results on the human immortalized keratinocyte (HaCaT) cell line are presented in Figure 1. Interestingly, turmeric NADES 6, 7, 13, and 15, which demonstrated the highest tyrosinase inhibitory activity, also exhibited cytotoxic effects at a concentration of 125 μg/mL on the HaCaT cell line. In contrast, NADES 14 (ChCl/lactic acid/water 1:2:5) extract showed no statistically significant cytotoxicity compared to untreated cells and emerged as one of the most promising tyrosinase inhibitors with minimal impact on HaCaT cells. These cytotoxic effects on keratinocytes can be attributed to the presence of citric acid in the NADES mixtures (6, 7, 13 and 15), as citric acid is known to induce apoptosis in HaCaT cells through caspase-dependent and mitochondrial signaling pathways [50]. Conversely, NADES 14, containing lactic acid, exhibits no statistically significant decrease in the viability of HaCaT cells while maintaining one of the highest levels of tyrosinase inhibitory activity, despite the reported antiproliferative effects of lactic acid on HaCaT cells [51]. Among the tested extracts, turmeric NADES 3 (ChCl/glycerol/water 1:2:5) extract demonstrated proliferative effects on HaCaT cells. These effects cannot be attributed to the NADES composition, phenolic content, or curcumin levels, suggesting the potential presence of a specific compound capable of promoting keratinocyte proliferation.

### 2.5. Inhibitory Effects of NADES Extracts on α-Amylase

*α*-amylase, an enzyme responsible for starch hydrolysis, is of interest in type 2 diabetes management due to its role in glucose release. Inhibition of *α*-amylase slows carbohydrate breakdown, reducing glucose absorption and helping to manage postprandial hyperglycemia [30]. Testing NADES turmeric extracts on *α*-amylase activity, allowed evaluation of their potential as natural inhibitors, possibly as alternatives or complements to synthetic antidiabetic drugs.

**Figure 2:**
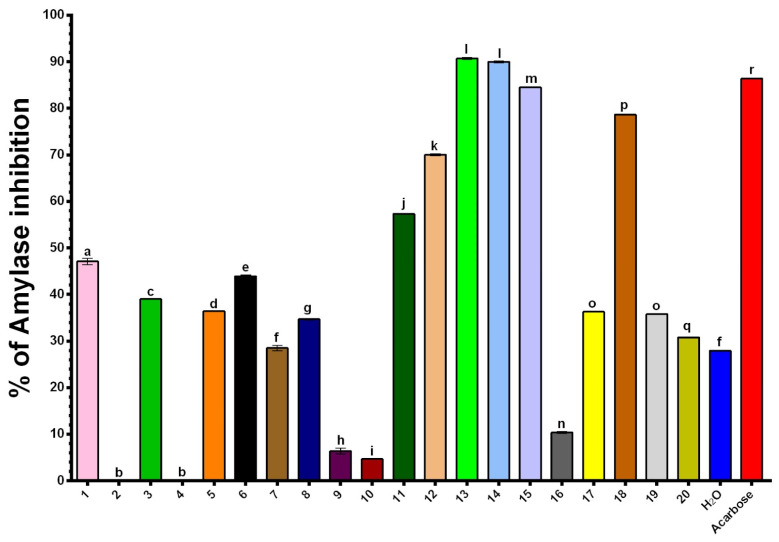
Percentages of inhibition for α-amylase. Distinct letters above the columns indicate statistically significant differences (*p* < 0.05) as determined by Tukey’s multiple comparisons test.

The inhibitory capacity of turmeric extracts against *α*-amylase was assessed at concentrations of 125 μg/mL, as shown in Figure 2 and Table S1 (Supplementary Materials). Extracts obtained using NADES 13 (ChCl/citric acid/water 1:2:3) and NADES 14 (ChCl/lactic acid/water 1:2:5) demonstrated the highest antidiabetic activity (90.7% and 90.0%, respectively), surpassing the inhibitory effect of acarbose, the positive control, and exhibiting an *α*-amylase inhibition potential three times greater than that of the aqueous extract (27.9%). NADES 15 (ChCl/citric acid/water 1:1:5) and NADES 18 (ChCl/1,2-propanediol/water 1:1:1) provided extracts that also exhibited notable activity. The results indicate that NADES extracts containing citric or lactic acid in their formulations show the most pronounced activity. Furthermore, an increase in the mole fraction of citric acid within the NADES formulation appears to enhance activity significantly. For example, NADES 13, with a 33.3% molar fraction of citric acid, displayed twice the inhibitory activity of NADES 6 (43.9%), which contains a 25% molar fraction of the acid (Figure 2). In addition, studies indicate that curcumin has moderate efficacy in inhibiting α-amylase and *α*-glucosidase activity, with certain curcumin derivatives showing enhanced potential in enzyme inhibition. However, due to limited data, further research on turmeric metabolites is required to fully understand their potential antidiabetic effects [31].

### 2.6. Curcumin Content in NADES Extracts

The medicinal properties of turmeric are primarily attributed to curcumin, a yellow-colored diarylheptanoid with well-documented bioactivities, including antioxidant, anti-inflammatory, antimicrobial, anticancer, neuroprotective, and cardioprotective effects [52]. These properties yield curcumin promising for therapeutic applications, particularly in the treatment and management of chronic diseases. Furthermore, curcumin is utilized in the food industry as a natural colorant and preservative due to its antimicrobial properties [53], and in the cosmetic industry for its antioxidant and anti-ageing benefits [54]. Traditionally, organic solvents such as ethanol, methanol, and acetone have been widely employed for curcumin extraction [55]. However, due to the toxicity of organic solvents, natural NADES have emerged as eco-friendly and non-toxic alternatives for the extraction of curcumin.

**Figure 3:**
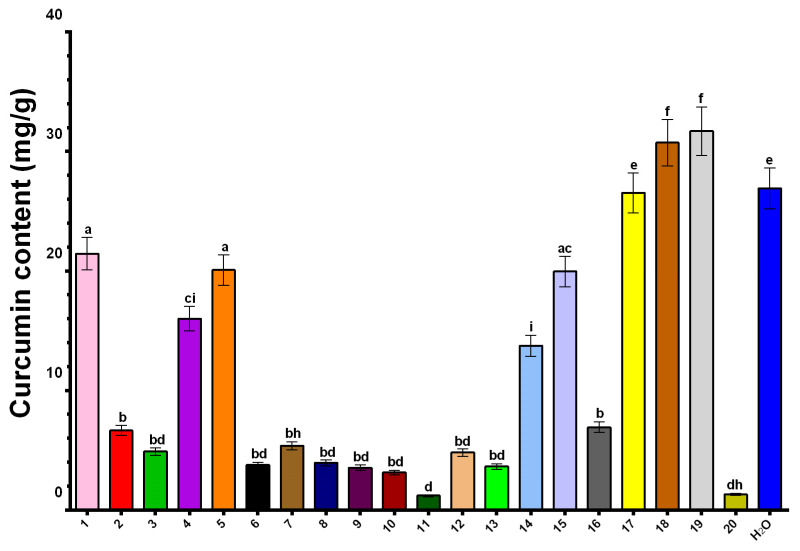
Curcumin content in NADES and water extracts. Distinct letters above the columns indicate statistically significant differences (*p* < 0.05) as determined by Tukey’s multiple comparisons test.

In this study, NADES formulations were evaluated for their curcumin extraction efficiency, expressed as milligrams of curcumin per gram of dry plant material. The results indicated significant variability among the extracts (Figure 3). The highest concentrations were observed in extracts obtained with NADES 19 (Glycerol/betaine/water 1:1:3) (31.70 mg/g) and NADES 18 (ChCl/1,2-propanediol/water 1:1:1) (30.73 mg/g), outperforming the aqueous extract (26.91 mg/g). Other notable formulations included NADES 17 (ChCl/glycerol/citric acid/water 0.5:2:0.5:5) (26.53 mg/g), NADES 1 (ChCl/glycerol/water 1:1:5) (21.45 mg/g), NADES 5 (ChCl/glycerol/water 1:1:2) (20.08 mg/g), and NADES 15 (ChCl/citric acid/water 1:1:5) (19.95 mg/g). Conversely, NADES 6 (ChCl/citric acid/water 1:2:5) (3.77 mg/g), NADES 7 (ChCl/citric acid/water 2:1:5) (5.38 mg/g), NADES 8 (ChCl/glycerol 1:1) (3.95 mg/g), and NADES 12 (ChCl/xylitol/water 1:1:5) (4.82 mg/g) exhibited low curcumin extraction capabilities, with NADES 9 (ChCl/glycerol 1:2) (3.54 mg/g), NADES 10 (ChCl/glycerol 1:3) (3.14 mg/g), and NADES 20 (glycerol/lysine/water 1:1:3) (1.30 mg/g) showing particularly poor performance (Table S2).

The highest curcumin concentrations were observed in NADES formulations containing betaine (NADES 19) or ChCl (NADES 18). These findings align with the study by Huber et al., which suggested that NADES components containing quaternary nitrogen may enhance curcumin solubility through cation–π interactions between the positively charged nitrogen and the extended π–system of curcumin [17]. Additionally, Koh et al. highlighted the superior extraction efficiency of NADES compared to water, as well as their ability to stabilize curcumin [27]. This stabilization was previously confirmed by Jelinski et al., who reported that curcumin degradation was absent in extracts obtained using a ChCl/glycerol NADES mixture, whereas a 5% degradation was observed in methanol extracts exposed to sunlight for two hours [56]. In addition, the extraction efficiency of NADES 18 (ChCl/1,2-propanediol/water 1:1:1), containing 33% water, and NADES 7, 8, and 9 (ChCl/glycerol in molar ratios of 1:1, 1:2, and 1:3, respectively), demonstrates that the presence of water as a component enhances the yield of curcumin. Notably, moderate extraction efficiency was observed for NADES 1 (ChCl/glycerol/water 1:1:5), NADES 14 (ChCl/lactic acid/water 1:2:5) and NADES 17 (ChCl/glycerol/citric acid/water 0.5:2:0.5:5) can be attributed to their elevated water content relative to other mixtures. On the other hand, NADES 18 (ChCl/1,2-propanediol/water 1:1:1) also stands out from other ChCl-based mixtures with water as one of its components due to the highest mole fraction for ChCl (33%). These findings suggest that increasing the mole fraction of ChCl or betaine—components capable of forming cation–π interactions with curcumin—and the mole fraction of water in the mixture enhances curcumin yield in the extract. Consequently, such mixtures highlight the potential of NADES as efficient and protective solvents for curcumin extraction.

## 3. Materials and Methods

### 3.1. Chemicals and Reagents

All chemicals and reagents utilized in this study were procured from reputable commercial suppliers. Ethanol and glycerol were sourced from ZORKA Pharma (Šabac, Serbia). The free radical 2,2-iphenyl-1-picrylhydrazyl (DPPH) was supplied by Fluka (Buchs, Switzerland). Phosphate buffer components (disodium phosphate and monosodium phosphate), as well as 2,2′-azino-bis(3-ethylbenzothiazoline-6-sulfonic acid) diammonium salt (ABTS), 6-hydroxy-2,5,7,8-tetramethylchroman-2-carboxylic acid (Trolox), protocatechuic acid (PCA), choline chloride, 1,2-propanediol, lactic acid, betaine, urea, and L-lysine, were purchased from Sigma-Aldrich (Darmstadt, Germany). Thermo Scientific (Waltham, MA, USA) sourced D-glucose, D-fructose, xylitol and citric acid. Ascorbic acid was obtained from Betahem (Belgrade, Serbia). In contrast, the Folin–Ciocalteu (FC) reagent, potassium persulfate, sodium carbonate, glycerol, 3,4-dihydroxy-L-phenylalanine (L-DOPA) and 3,5-dinitro salicylic acid (DNSA) were sourced from Merck (Darmstadt, Germany). Tripton LP0042 and yeast extract LP0021 were supplied by Oxoid LTD (Basingstoke, UK) and nutrient agar was provided by Lab M (Bury, UK). Phosphate-buffered saline (PBS, 1X, pH 7.4) was sourced from UFC Biotechnology (Amherst, NY, USA). Several other reagents, including streptomycin, sodium chloride, disodium phosphate, monosodium phosphate, mushroom-derived tyrosinase enzyme, kojic acid, α-amylase from porcine pancreas, and acarbose were purchased from Sigma-Aldrich Chemie GmbH (Steinheim, Germany).

### 3.2. Preparation of NADESs

*C. longa* was bought in the local store and NADES were prepared using a heating and stirring method. Specific molar ratios of HBAs and HBDs were accurately weighed, and 20% *w*/*w* water was added to reduce viscosity. The mixtures were then stirred continuously with a magnetic stirrer in an oil bath at 80 °C until clear, homogenous solvents were formed. The compositions of the NADES used in this study are detailed in Table 3.

### 3.3. Extractions

For the extraction process, the components of the eutectic mixture were first combined and heated at 80 °C using a magnetic stirrer until a clear, homogenous phase was formed. Afterward, a specific amount of water was added, and the mixture was stirred for an additional 30 min. Turmeric powder was used as the raw material for preparing the extracts, with 20 different NADES solvents serving as the extraction media. To ensure consistency, the turmeric powder was further ground using a home miller. For the extraction process, 10 mL of each NADES or water was transferred into an Erlenmeyer flask. The flask was then placed on a magnetic stirrer preheated to 50 °C to facilitate the extraction. A total of 1 g of turmeric powder was added, and stirring was continued for an additional 30 min while maintaining a constant temperature of 50 °C. Once the extraction was complete, the mixture was cooled to room temperature and 5 mL of each extract was transferred to a 50 mL vial and diluted with 20 mM sodium phosphate buffer (pH 7.45) to a final volume of 50 mL. The diluted extracts were centrifuged at 9000 rpm for 10 min, and the supernatant was carefully separated from the residue using a Pasteur pipette. The diluted extracts were then stored in the freezer in labeled 50 mL vials, with labels 1–20 corresponding to the specific NADES used (as indicated in Table 3).

### 3.4. FTIR (Fourier-Transform Infrared) Spectroscopy

Absorbance spectra of the NADES components and NADES mixtures were recorded using a Nicolet iS20 FTIR equipped with a Smart iTX Diamond ATR accessory (Thermo Fisher Scientific Inc., Waltham, MA, USA). Spectral data were collected over the 4000 to 650 cm^−1^ range, with a resolution of 4 cm^−1^, and 64 scans were performed per sample. A few drops of concentrated NADES sample were applied to the ATR crystal, and after solvent evaporation, the FTIR spectrum was recorded. Each sample spectrum was corrected using an air background spectrum.

### 3.5. Spectrophotometric Assays

Spectrophotometric measurements were performed using a GBC UV–visible Cintra 6 spectrophotometer (Dandenong, VIC, Australia) with each assay conducted in triplicate. To choose the most effective NADES for extracting bioactives, various factors were considered, including the results of antioxidative capacity assays (DPPH and ABTS) and the total phenol content (TPC) to identify the optimal solvent for maximizing extract potency.

#### 3.5.1. Total Phenolic Content (TPC)

The total phenolic content (TPC) was quantified using the Folin–Ciocalteu method [57,58]. A volume of 0.5 mL of each extract was diluted with 0.5 mL of distilled water and combined with 2.5 mL of a 10% (*w*/*v*) Folin–Ciocalteu reagent. After a 5 min incubation period, 2.0 mL of a 7.5% (*w*/*w*) sodium carbonate solution was added. The mixtures were then incubated for 2 h at room temperature. Absorbance was measured at 765 nm, using gallic acid as the reference standard (20–120 mg/L) to construct the calibration curve (y = 0.01x − 0.0322, *R*^2^ = 0.997). The TPC results were reported as milligrams of gallic acid equivalents (GAE) per g of dry sample.

#### 3.5.2. DPPH Radical Scavenging Assay

The radical scavenging activity (RSA) was assessed using the DPPH assay [59]. In this procedure, 0.1 mL of each extract was mixed with 4 mL of a 79 μmol/L DPPH solution in methanol. Following a 1 h incubation at room temperature in the dark, the absorbance was recorded at 517 nm. Trolox served as the reference standard, with a concentration range of 100–600 μmol/L to construct the calibration curve (y = 0.0005x + 0.0088, *R*^2^ = 0.999). The RSA values were expressed as micromoles of Trolox equivalents (TE) per gram of dry sample.

#### 3.5.3. ABTS Radical Scavenging Assay

The ABTS•^+^ radical cation reduction assay was employed to evaluate the radical scavenging capacity of the extracts [59,60]. ABTS•^+^ solution was prepared by mixing 7 mmol/L ABTS solution with 245 mmol/L potassium persulfate, resulting in a final concentration of 2.45 mmol/L potassium persulfate. This solution was stored in the dark at room temperature for 16 h. After dilution, the reaction solution was adjusted to an absorbance of 0.7028 at 734 nm. A total of 10 μL of the diluted herbal extract was added to 190 μL of the ABTS•^+^ solution, and absorbance was measured immediately at 734 nm using a BioTek 800 TS spectrophotometer. Trolox standard solutions (10–500 μmol/L) were used to construct the calibration curve (y = 0.0013x − 0.0054, *R*^2^ = 0.997). Results were presented as the percentage inhibition of the ABTS radical and as micromoles of Trolox equivalents per gram of dry weight (mmol TE/g).

### 3.6. Agar Well Diffusion Test

The antibacterial activity of the extracts was assessed using an agar well diffusion assay against *Listeria monocytogenes* ATCC 13932 and *Salmonella typhimurium* ATCC 14028, following the previously described procedure by Lazović et al. [29] with modifications. Bacterial cells were cultivated in Luria–Bertani (LB) broth to an optical density (OD) of approximately 0.5, monitored at 600 nm using a CINTRA 6 UV–Vis spectrophotometer (GBC Scientific Equipment Ltd., Dandenong, VIC, Australia). The LB medium was prepared with 10 g tryptone, 5 g yeast extract, and 5 g NaCl per liter of distilled water and autoclaved for 15 min at 121 °C. Bacterial suspensions were mixed with nutrient agar at a 1:50 (*v*/*v*) ratio and poured into petri dishes. Turmeric extracts, diluted in PBS (1:5 *v*/*v*), were added (100 μL) to 10 mm diameter wells in the inoculated agar. Plates were incubated at 37 °C for 24 h. Streptomycin (5 mg/mL in PBS) was a positive control, while PBS alone was a negative control. All experiments were conducted in triplicate.

### 3.7. Assessing of Skin-Related Effects

#### 3.7.1. Tyrosinase Inhibition Assay

The inhibitory activity of NADES extracts against tyrosinase was assessed spectrophotometrically in a 96-well microtiter plate using a BioTek 800 TS spectrophotometer (Agilent Technologies, Inc., Santa Clara, CA, USA), following the method of Ivković et al. using L-DOPA as the substrate [14]. For all enzyme assays, a concentration of 125 µg of powdered plant material per mL of NADES was used. Phosphate buffer (pH 6.8; 20 mmol/L) was added (140 µL) to 20 µL of plant extract (diluted in buffer) and 20 µL of enzyme (750 IU/mL). After 20 min of incubation at 37 °C, 20 µL of 5 mmol/L L-DOPA was added, resulting in a brown coloration. Absorbance at 475 nm was measured, and inhibition (%) was calculated as:

$$I\left(\%\right)=100\times \frac{\left[\left(A-B\right)-\left(C-D\right)\right]}{\left[A-B\right]}$$

where A = negative sample control, B = blank, C = positive sample control, and D = negative enzyme control. Kojic acid (125 μg/mL) was used as a positive control.

#### 3.7.2. Keratinocyte Viability Assessment

All HaCaT keratinocytes (AddexBio, San Diego, CA, USA, T0020001) were cultured as monolayers in Dulbecco’s Modified Eagle Medium (Gibco, Grand Island, NY, USA) supplemented with 10% fetal bovine serum, 2 mM glutamine, 100 IU/mL penicillin, 0.1 mg/mL streptomycin, 1% nonessential amino acid solution, and 1 mM sodium pyruvate. Cells were maintained at 37 °C in 5% CO_2_.

HaCaT cells (10,000 cells/well) were seeded in 96-well plates. After 24 h of adherence, turmeric NADES extracts were added in concentrations of 125 μg of powdered plant material per mL of NADES, with control wells where cells were maintained only in the medium. Experiments were performed in triplicate. Cell viability was assessed using the MTT assay, adapted from Mosmann (1983) [59]. After 24 h of treatment, 20 µL of MTT solution (5 mg/mL in PBS) was added to each well and incubated for 2 h at 37 °C. Formazan crystals were dissolved with 200 µL DMSO, and absorbance was measured at 570 nm (test) and 630 nm (reference). Viability was expressed as a percentage relative to untreated control cells.

### 3.8. α-Amylase Inhibition Assay

The inhibitory activity of NADES extracts against *α*-amylase was evaluated spectrophotometrically, following the methodology described by Kifle and Enyew [60]. In 15 mL plastic vials, 50 µL of turmeric extract solution was combined with 25 µL of 1% (*w*/*v*) starch solution and incubated for 10 min at 25 °C. Subsequently, 25 μL of *α*-amylase (0.5 mg/mL) was added, and the mixture was incubated for an additional 10 min at 25 °C. Afterwards, 100 μL of 3,5-dinitrosalicylic acid (DNSA) reagent was added, and the solution was incubated for 5 min at 100 °C. The resulting solution was diluted with 1 mL of distilled water, and absorbance was measured at 540 nm using a CINTRA-6 UV–Vis spectrophotometer. Acarbose (125 μg/mL) served as the standard, while a blank sample was prepared by substituting distilled water for the turmeric extract.

### 3.9. LC–MS Analysis of Curcumin

LC–MS (Thermo Scientific™ Vanquish™ Core HPLC system coupled to the Orbitrap Exploris 120 mass spectrometer, San Jose, CA, USA) was used to determine curcumin content in the investigated extracts. The liquid chromatography system was equipped with a Hypersil GOLD™ C18 analytical column (50 × 2.1 mm, 1.9 μm particle size), thermostated at 40 °C. The mobile phase consisted of ultrapure water with 0.1% formic acid (A) and acetonitrile with 0.1% formic acid (B), using the following gradient: 5% B in the first minute, 5–95% B from 1 to 10 min, 95% B from 10 to 12 min, and 5% B until 15 min. The flow rate was set at 300 μL/min, and the injection volume was 5 μL. The Orbitrap Exploris 120 mass spectrometer was equipped with an ESI source operating in negative ionization mode, with full scan MS monitored from 100 to 1500 *m*/*z* at a resolution of 60,000 FWHM. The other LC and MS parameters are previously described in Stojković et al., 2024 [61]. Data acquisition was carried out using the Xcalibur^®^ data system (Thermo Finnigan, San Jose, CA, USA). Curcumin was identified and quantified based on its spectral characteristics, including mass spectra, accurate mass, and characteristic fragmentation.

### 3.10. Statistical Analysis

Data are expressed as mean ± standard deviation and analyzed using the GraphPad Prism 6 software with Tukey’s multiple comparison post-tests (GraphPad, San Diego, CA, USA). All incubations were conducted in triplicate, except for the LC–MS analysis of curcumin content, where each sample was analyzed in duplicate with two biological replicates. Statistical differences were evaluated using one-way ANOVA, with significance set at *p* < 0.05.

## 4. Conclusions

This study demonstrates the superior efficiency of several NADES compared to conventional solvents, such as water, for extracting bioactive compounds from turmeric powder. Specific NADES formulations achieved higher yields of curcumin and phenolics while preserving antioxidant and enzyme inhibitory activities, highlighting their potential for applications across various industries.

Among the tested formulations, NADES 2 (ChCl/fructose/water 1:1:5) was the most effective for extracting total phenolics, outperforming water. Additionally, NADES 17 (ChCl/glycerol/citric acid/water 0.5:2:0.5:5) and NADES 20 (glycerol/lysine/water 1:1:3) exhibited superior antioxidant activities in DPPH assay, while NADES 11 (ChCl/urea/water 1:2:5) exhibited the highest activity in ABTS assay. NADES 14 (ChCl/lactic acid/water 1:2:5) showed the strongest tyrosinase inhibition, suggesting its potential for skin-brightening applications, while NADES 13 and 15 (ChCl/citric acid/water 1:2:3 and 1:1:5, respectively) and NADES 14 exhibited significant α-amylase inhibition, surpassing the reference compound acarbose, and indicating the potential for managing hyperglycemia.

Overall, NADES formulations, including NADES 13, 14, and 15, demonstrated dual enzyme inhibitory activities and antioxidant properties, positioning them as promising candidates for the cosmetic, food, and pharmaceutical industries. Among all formulations, NADES 15 (ChCl/citric acid/water 1:2:5) emerged as the most versatile, showing performance comparable to or exceeding aqueous extracts in most evaluations.

In conclusion, this study establishes NADES as efficient and sustainable alternatives to conventional solvents for extracting bioactive compounds. Their customizable composition offers tailored extraction efficiency for various applications. Future research should focus on optimizing extraction parameters, assessing the stability of NADES-derived extracts, and expanding their application to other plant materials and bioactive compounds. These findings support the development of sustainable and effective methods for bioactive compound extraction, aligning with the principles of green chemistry.

## Figures and Tables

**Table 1:** Total phenolic content (TPC) and radical scavenging activities against DPPH• and ABTS•^+^ in NADES and water turmeric extract (mean values for duplicates ± standard deviation). Distinct letters next to the corresponding values indicate statistically significant differences (*p* < 0.05) as determined by Tukey’s multiple comparisons test.

Extract	NADES Composition	TPC (mg GAE/g)	DPPH (μmol TE/g)	ABTS (μmol TE/g)
NADES 1	ChCl/Glycerol/Water 1:1:5	12.42 ± 0.13 ^a^	6.9 ± 0.5 ^ae^	0.49 ± 0.03 ^ad^
NADES 2	ChCl/Fructose/Water 1:1:5	54.77 ± 0.22 ^b^	8.5 ± 1.6 ^ae^	0.34 ± 0.02 ^ad^
NADES 3	ChCl/Glycerol/Water 1:2:5	13.97 ± 0.44 ^af^	4.88 ± 0.04 ^a^	4.25 ± 0.08 ^bf^
NADES 4	ChCl/Glucose/Water 1:1:3	6.92 ± 0.14 ^c^	17.4 ± 0.9 ^bcf^	4.20 ± 0.16 ^bf^
NADES 5	ChCl/Glycerol/Water 1:1:2	13.45 ± 0.36 ^af^	19.82 ± 1.02 ^cb^	4.29 ± 0.02 ^bf^
NADES 6	ChCl/Citric acid/Water 1:2:5	14.08 ± 0.09 ^af^	20.2 ± 3.2 ^bf^	3.94 ± 0.11 ^ce^
NADES 7	ChCl/Citric acid/Water 2:1:5	8.25 ± 0.10 ^cd^	20.4 ± 4.5 ^bcg^	4.18 ± 0.22 ^bf^
NADES 8	ChCl/Glycerol 1:1	9.41 ± 0.59 ^d^	7.6 ± 0.2 ^ae^	0.34 ± 0.23 ^ad^
NADES 9	ChCl/Glycerol 1:2	17.76 ± 0.17 ^e^	7.9 ± 2.9 ^ae^	4.09 ± 0.05 ^bf^
NADES 10	ChCl/Glycerol 1:3	14.88 ± 0.07 ^f^	8.6 ± 0.8 ^ae^	0.50 ± 0.04 ^ad^
NADES 11	Glycerol/Urea/Water 1:1:2	20.42 ± 0.06 ^g^	24.1 ± 0.6 ^cg^	4.74 ± 0.14 ^b^
NADES 12	ChCl/Xylitol/Water 1:1:5	12.74 ± 0.04 ^ak^	15.6 ± 3.8 ^bf^	1.65 ± 0.47 ^cd^
NADES 13	ChCl/Citric acid/Water 1:2:3	11.55 ± 0.92 ^k^	15.0 ± 0.5 ^bf^	3.45 ± 0.21 ^cefg^
NADES 14	ChCl/Lactic acid/Water 1:2:5	21.54 ± 0.19 ^gh^	16.4 ± 1.7 ^bf^	0.22 ± 0.05 ^ad^
NADES 15	ChCl/Citric acid/Water 1:1:5	15.75 ± 0.02 ^f^	22.1 ± 0.2 ^cg^	3.51 ± 0.19 ^cefg^
NADES 16	ChCl/Urea/Water 1:2:5	18.02 ± 0.09 ^e^	17.7 ± 0.2 ^bf^	3.00 ± 0.02 ^e^
NADES 17	ChCl/Glycerol/Citric acid/Water 0.5:2:0.5:5	20.36 ± 0.06 ^g^	30.5 ± 1.0 ^d^	4.62 ± 0.07 ^b^
NADES 18	ChCl/1,2-propanediol/Water 1:1:1	17.11 ± 0.01^e^	11.7 ± 0.8 ^ef^	1.04 ± 0.03 ^d^
NADES 19	Glycerol/Betaine/Water 1:1:3	22.80 ± 0.18 ^hj^	11.3 ± 0.3 ^af^	4.35 ± 0.03 ^bg^
NADES 20	Glycerol/Lysine/Water 1:1:3	36.84 ± 0.13 ^i^	29.6 ± 0.3 ^d^	4.64 ± 0.03 ^b^
H_2_O		24.07 ± 1.68 ^j^	26.6 ± 0.3 ^dg^	4.18 ± 0.89 ^bg^

**Table 2:** The antibacterial activity of turmeric extracts evaluated by the agar well diffusion assay presented as mean inhibition zone diameter (mm) ± standard deviation, based on triplicate measurement.

Extract	NADES Composition	*S. typhimurium* ATCC 14028	*L. monocytogenes* ATCC 13932
NADES 1	ChCl/Glycerol/Water 1:1:5	–	–
NADES 2	ChCl/Fructose/Water 1:1:5	–	–
NADES 3	ChCl/Glycerol/Water 1:2:5	–	–
NADES 4	ChCl/Glucose/Water 1:1:3	23.5 ± 1.5	–
NADES 5	ChCl/Glycerol/Water 1:1:2	–	–
NADES 6	ChCl/Citric acid/Water 1:2:5	27.0 ± 1.0	35.0 ± 1.0
NADES 7	ChCl/Citric acid/Water 2:1:5	24.5 ± 1.0	35.0 ± 1.0
NADES 8	ChCl/Glycerol 1:1	–	–
NADES 9	ChCl/Glycerol 1:2	–	–
NADES 10	ChCl/Glycerol 1:3	–	–
NADES 11	Glycerol/Urea/Water 1:1:2	–	11.0 ± 0.0
NADES 12	ChCl/Xylitol/Water 1:1:5	–	12.0 ± 0.5
NADES 13	ChCl/Citric acid/Water 1:2:3	25.0 ± 1.0	34.0 ± 2.0
NADES 14	ChCl/Lactic acid/Water 1:2:5	–	–
NADES 15	ChCl/Citric acid/Water 1:1:5	21.0 ± 1.5	35.0 ± 1.0
NADES 16	ChCl/Urea/Water 1:2:5	–	11.0 ± 0.0
NADES 17	ChCl/Glycerol/Citric acid/Water 0.5:2:0.5:5	15.0 ± 0.5	33.0 ± 1.0
NADES 18	ChCl/1,2-propanediol/Water 1:1:1	–	12.5 ± 0.5
NADES 19	Glycerol/Betaine/Water 1:1:3	–	12.0 ± 1.0
NADES 20	Glycerol/Lysine/Water 1:1:3	–	–
H_2_O		–	–
Streptomycin		23.0 ± 1.0	26.5 ± 1.5

**Table 3:** The compositions of the studied NADESs and their abbreviations should be placed in the main text near the first time they are cited.

Abbreviation	NADES Composition	Molar Ratio
NADES 1	ChCl/Glycerol/Water	1:1:5
NADES 2	ChCl/Fructose/Water	1:1:5
NADES 3	ChCl/Glycerol/Water	1:2:5
NADES 4	ChCl/Glucose/Water	1:1:3
NADES 5	ChCl/Glycerol/Water	1:1:2
NADES 6	ChCl/Citric acid/Water	1:2:5
NADES 7	ChCl/Citric acid/Water	2:1:5
NADES 8	ChCl/Glycerol	1:1
NADES 9	ChCl/Glycerol	1:2
NADES 10	ChCl/Glycerol	1:3
NADES 11	Glycerol/urea/Water	1:1:2
NADES 12	ChCl/Xylitol/Water	1:1:5
NADES 13	ChCl/Citric acid/Water	1:2:3
NADES 14	ChCl/Lactic acid/Water	1:2:5
NADES 15	ChCl/Citric acid/Water	1:1:5
NADES 16	ChCl/Urea/Water	1:2:5
NADES 17	ChCl/Glycerol/Citric acid/Water	0.5:2:0.5:5
NADES 18	ChCl/1,2-propanediol/Water	1:1:1
NADES 19	Glycerol/Betaine/Water	1:1:3
NADES 20	Glycerol/Lysine/Water	1:1:3

## Data Availability

Data are contained within the article and Supplementary Materials.

## Supplementary Materials

The following supporting information can be downloaded at: https://www.mdpi.com/article/10.3390/plants14020163/s1, Figure S1: FTIR spectra of NADES components: (a) Cholin chloride (ChCl), (b) Glycerol, (c) 1,2-Propanediol, (d) Fructose, (e) Glucose, (f) Xylitol, (g) Lactic acid, (h) Citric acid, (i) Urea, (j) Betaine, (k) Lysine; Figure S2: FTIR spectra of NADES mixtures: (a) ChCl/Glycerol/Water 1:1:5 (NADES 1); (b) ChCl/Glycerol/Water 1:2:5 (NADES 3); (c) ChCl/Glycerol/Water 1:1:2 (NADES 5); (d) ChCl/1,2-propanediol/Water 1:1:1 (NADES 18); (e) ChCl/Glycerol 1:1 (NADES 8); (f) ChCl/Glycerol 1:2 (NADES 9); (g) ChCl/Glycerol 1:3 (NADES 10); (h) ChCl/Fructose/Water 1:1:5 (NADES 2); (i) ChCl/Glucose/Water 1:1:3 (NADES 4); (j) ChCl/Xylitol/Water 1:1:5 (NADES 12); (k) ChCl/Lactic acid/Water 1:2:5 (NADES 14); (l) ChCl/Citric acid/Water 1:2:5 (NADES 6); (m) ChCl/Citric acid/Water 2:1:5 (NADES 7); (n) ChCl/Citric acid/Water 1:2:3 (NADES 13); (o) ChCl/Citric acid/Water 1:1:5 (NADES 15); (p) ChCl/Glycerol/Citric acid/Water 0,5:2:0,5:5 (NADES 17); (q) ChCl/Urea/Water 1:2:5 (NADES 16); (r) Glycerol/Urea/Water 1:1:2 (NADES 11); (s) Glycerol/Betaine/Water 1:1:3 (NADES 19); (t) Glycerol/Lysine/Water 1:1:3 (NADES 20); Table S1: Percentages of inhibition for tyrosinase and α-amylase. Kojic acid served as the standard for the tyrosinase inhibition assay, while acarbose was used as the reference standard for the α-amylase inhibition assay; Table S2: Curcumin content expressed as mg/g of dry plant sample ± standard deviation.
