# Supplementary material for: Green Extraction of Bioactives from Curcuma longa Using Natural Deep Eutectic Solvents: Unlocking Antioxidative, Antimicrobial, Antidiabetic, and Skin Depigmentation Potentials
Source: Plants (Basel). 2025 Jan 8;14(2):163. doi: 10.3390/plants14020163 (PMC11768996; doi:10.3390/plants14020163)
Supplement: Supplementary file 1 [file plants-14-00163-s001.zip › plants-3374056-supplementary.pdf]

Supplementary material

Green extraction of bioactives from *Curcuma longa* using natural deep eutectic solvents: unlocking antioxidative, antimicrobial, antidiabetic, and skin depigmentation potentials

Jelena Jovanović [1,†], Marko Jović [2,†], Jelena Trifković [3], Katarina Smiljanić [4], Uroš Gašić [5], Maja Krstić Ristivojević [4] and Petar Ristivojević [3,*]

[1] *Vinča Institute of Nuclear Sciences – National Institute of the Republic of Serbia, University of Belgrade, Mike Petrovića Alasa 12–14, 11001 Belgrade, Serbia;* jelena.jovanovic@vin.bg.ac.rs
[2] *Innovative Centre of the Faculty of Chemistry, Ltd., University of Belgrade-Faculty of Chemistry, Studentski Trg 12-16, 11158 Belgrade, Serbia* 2; markojovic@chem.bg.ac.rs
[3] *University of Belgrade - Faculty of Chemistry, Department of Analytical Chemistry, Studentski trg 12-16, 11158 Belgrade, Serbia;* jvelicko@chem.bg.ac.rs
[4] *University of Belgrade - Faculty of Chemistry, Centre of Excellence for Molecular Food Sciences and Department of Biochemistry, Studentski trg 12-16, 11158 Belgrade, Serbia;* katarinas@chem.bg.ac.rs; krstic_maja@chem.bg.ac.rs
[5] *University of Belgrade, Institute for Biological Research "Siniša Stanković" - National Institute of Republic of Serbia, Department of Plant Physiology, Bulevar despota Stefana 142, 11108 Belgrade, Serbia;* uros.gasic@ibiss.bg.ac.rs
**Correspondence: ristivojevic@chem.bg.ac.rs (P.M.R.)*
† These authors contributed to the work equally and should be regarded as co-first authors.

a) Cholin chloride

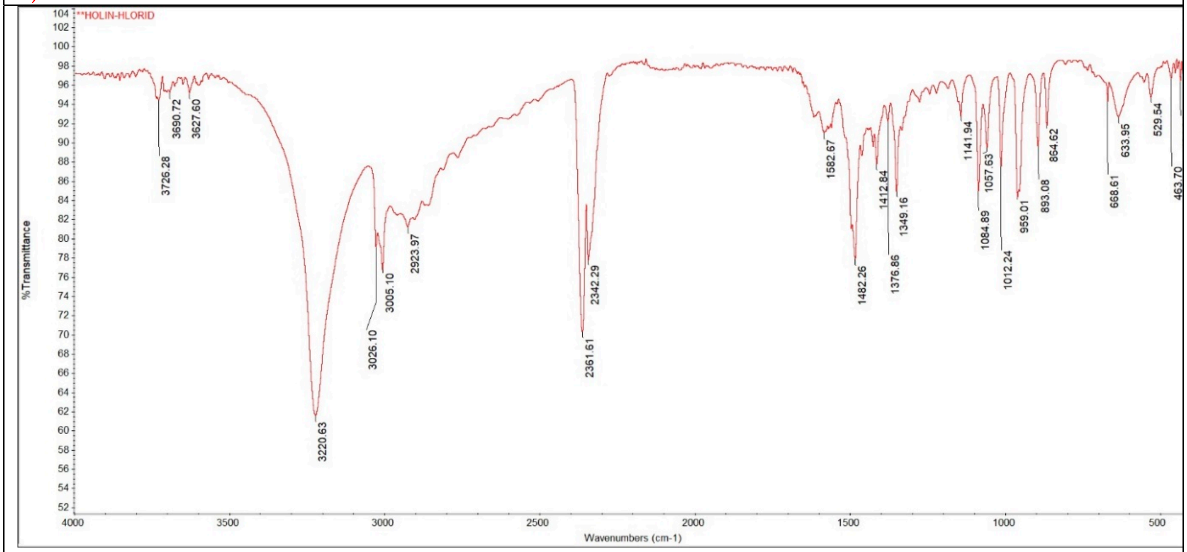

b) Glycerol

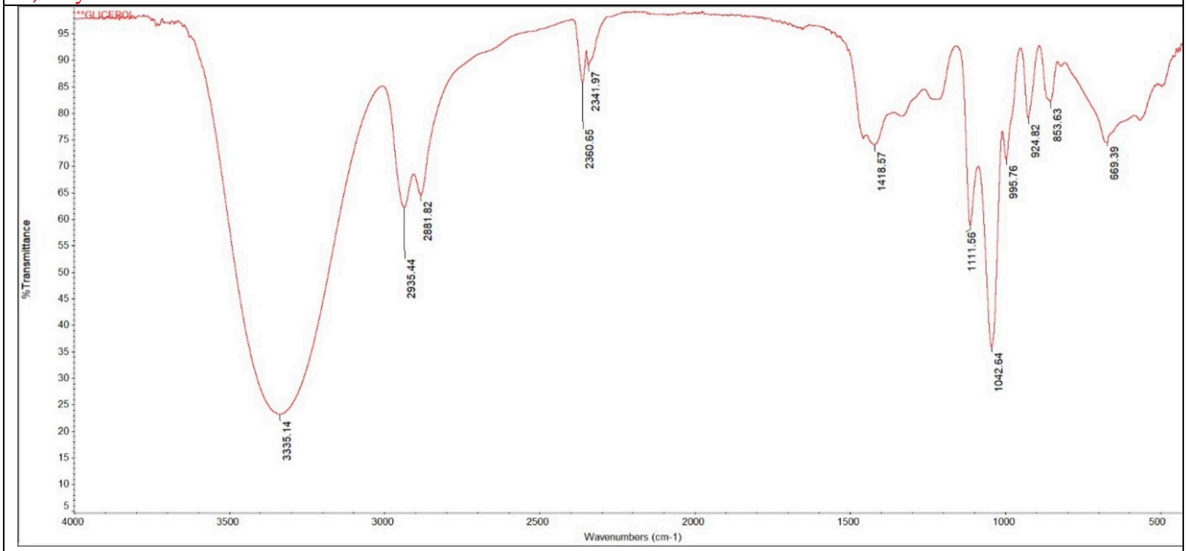

c) 1,2-Propanediol

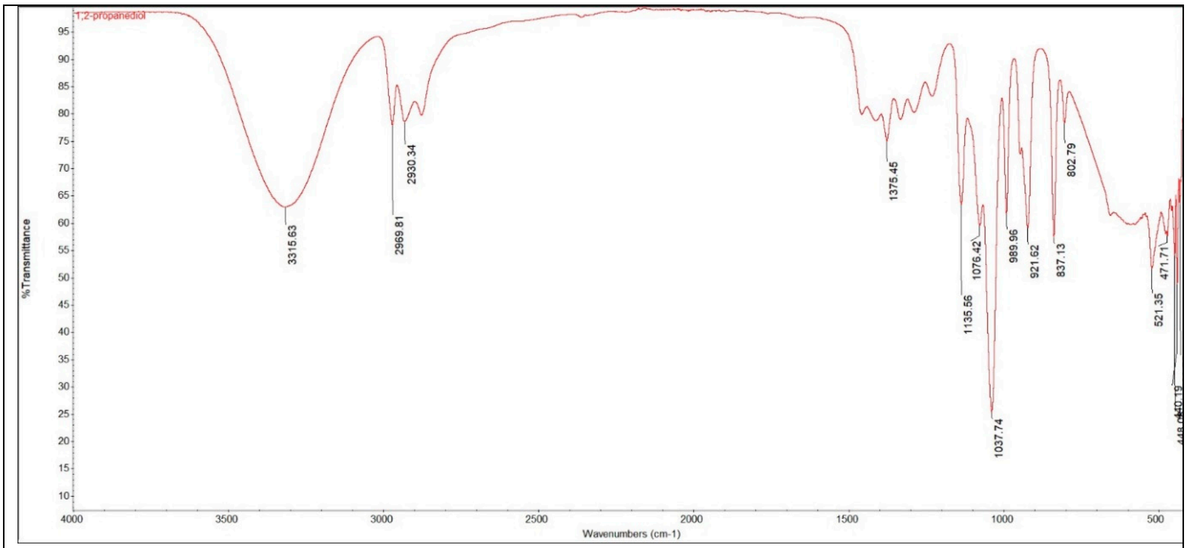

1,2-propanediol
%Transmittance
Wavenumbers (cm-1)
3315.63
2969.81
2930.34
1375.45
1135.56
1076.42
1037.74
989.96
921.62
837.13
802.79
521.35
471.71

d) Fructose

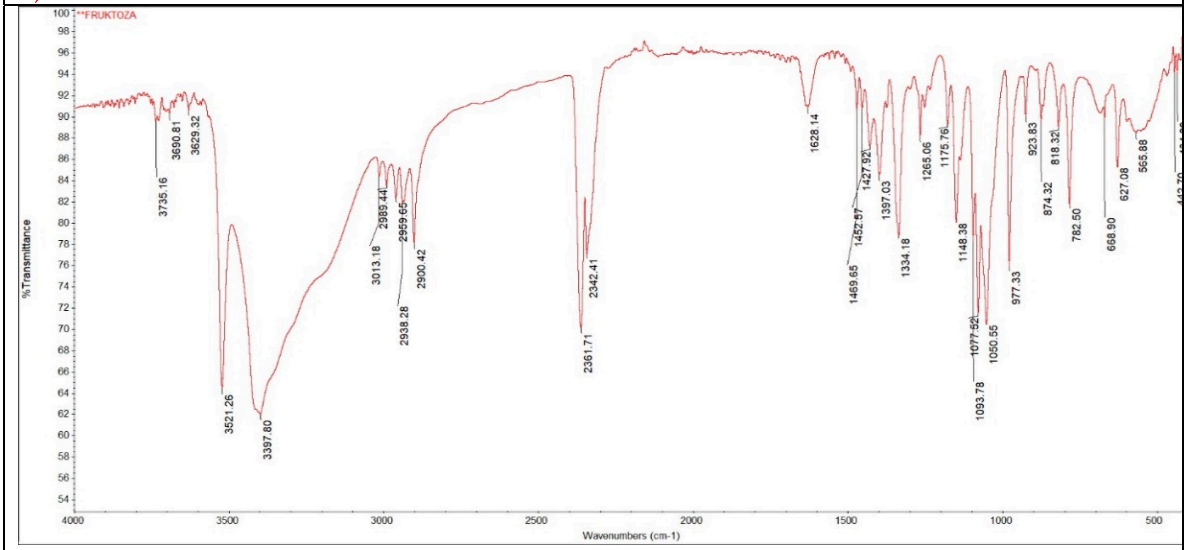

**FRUKTOZA
%Transmittance
Wavenumbers (cm-1)
3735.16
3690.81
3629.32
3521.26
3397.80
3013.18
2989.44
2959.65
2938.28
2900.42
2361.71
2342.41
1628.14
1469.65
1452.57
1427.92
1397.03
1334.18
1265.06
1175.76
1148.38
1093.78
1077.52
1050.55
977.33
923.83
874.32
818.32
782.50
668.90
627.08
565.88

e) Glucose

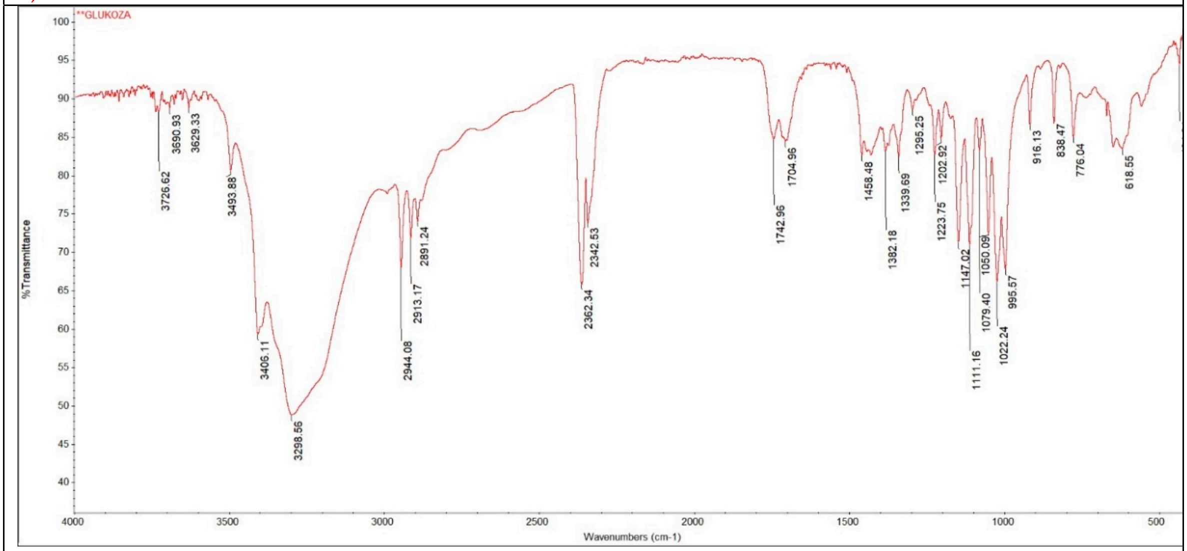

f) Xylitol

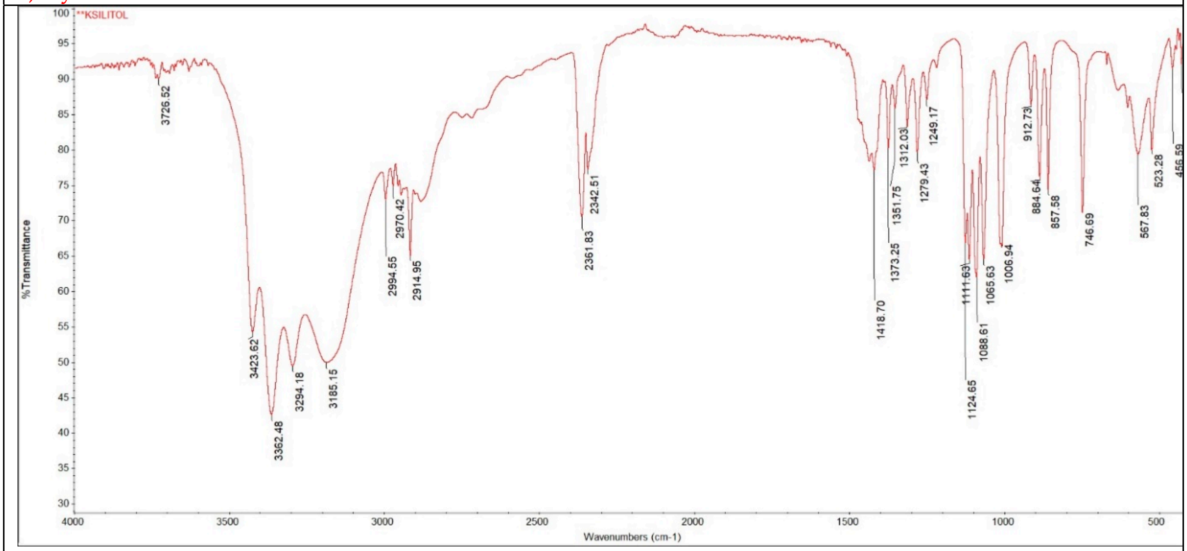

g) Lactic acid

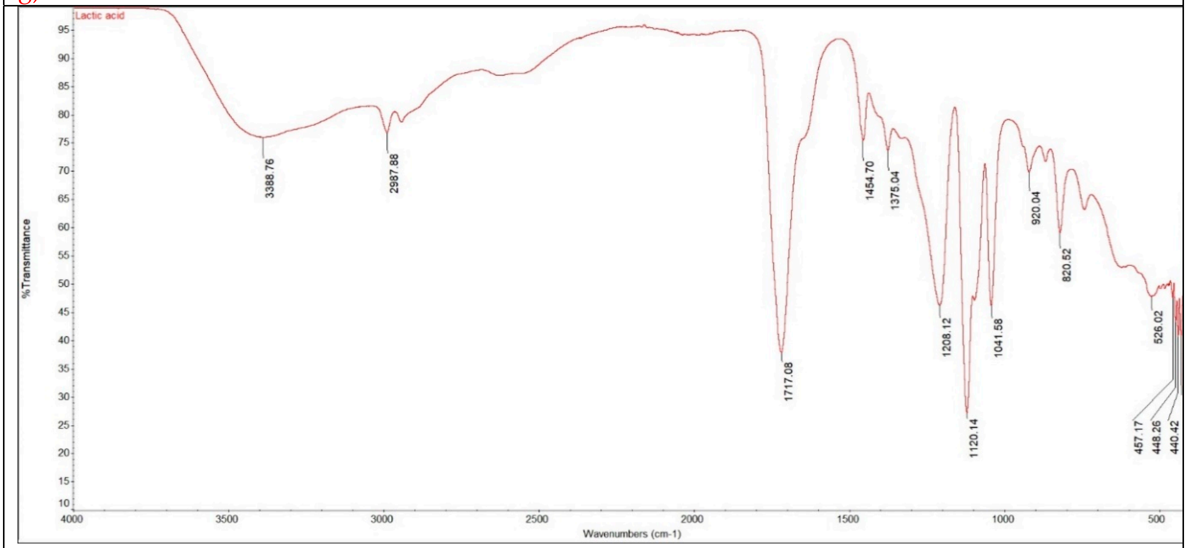

h) Citric acid

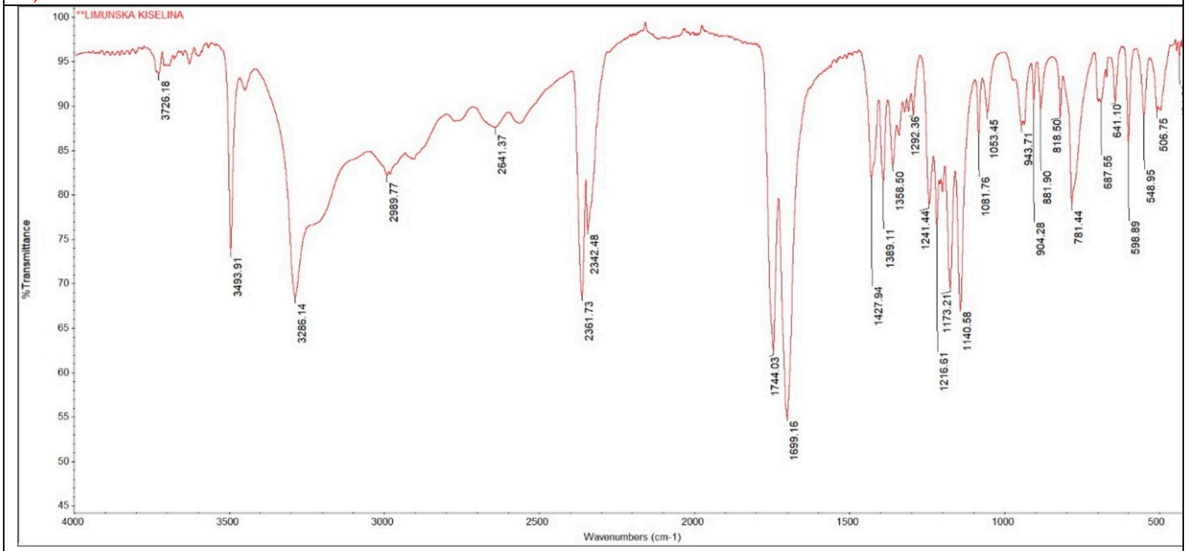

i) Urea

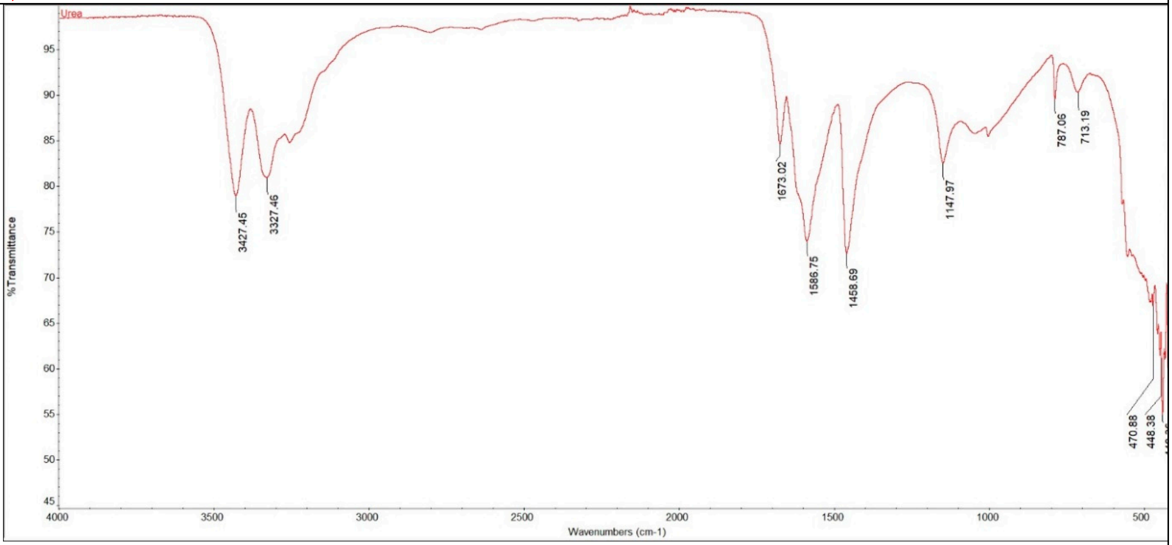

j) Betaine

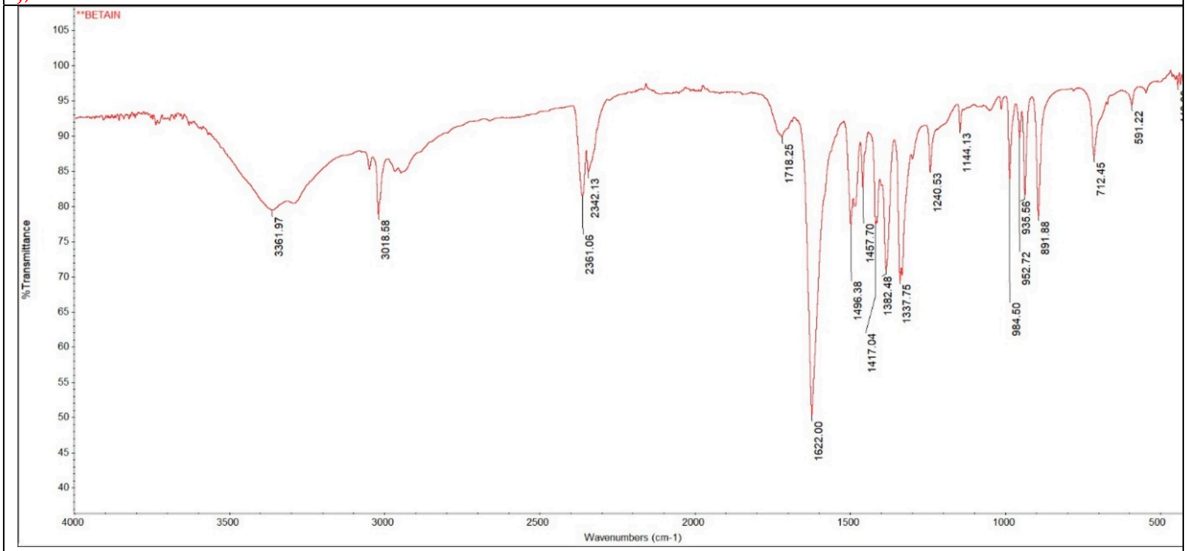

k) L-lysine

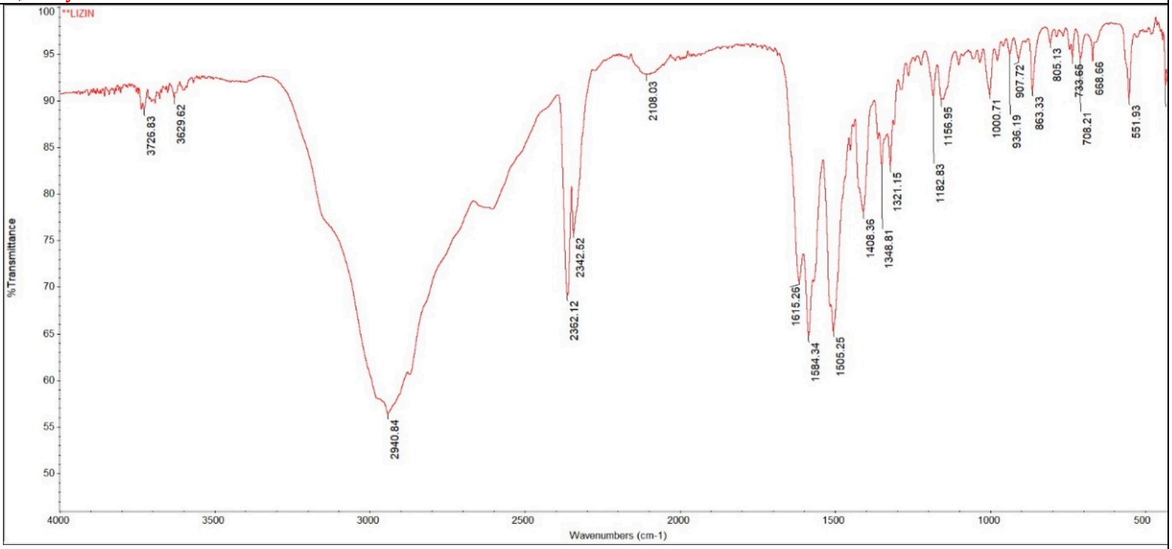

Figure S1. FTIR spectra of NADES components: a) Cholin chloride (ChCl), b) Glycerol, c) 1,2-Propanediol, d) Fructose, e) Glucose, f) Xylitol, g) Lactic acid, h) Citric acid, i) Urea, j) Betaine, k) Lysine.

a) ChCl/Glycerol/Water 1:1:5 (NADES 1)

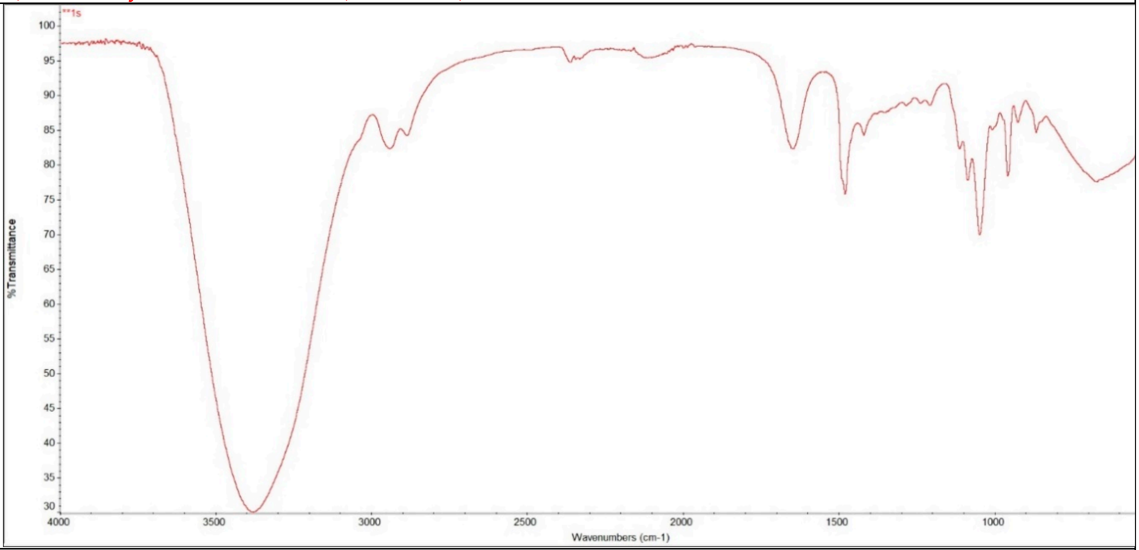

b) ChCl/Glycerol/Water 1:2:5 (NADES 3)

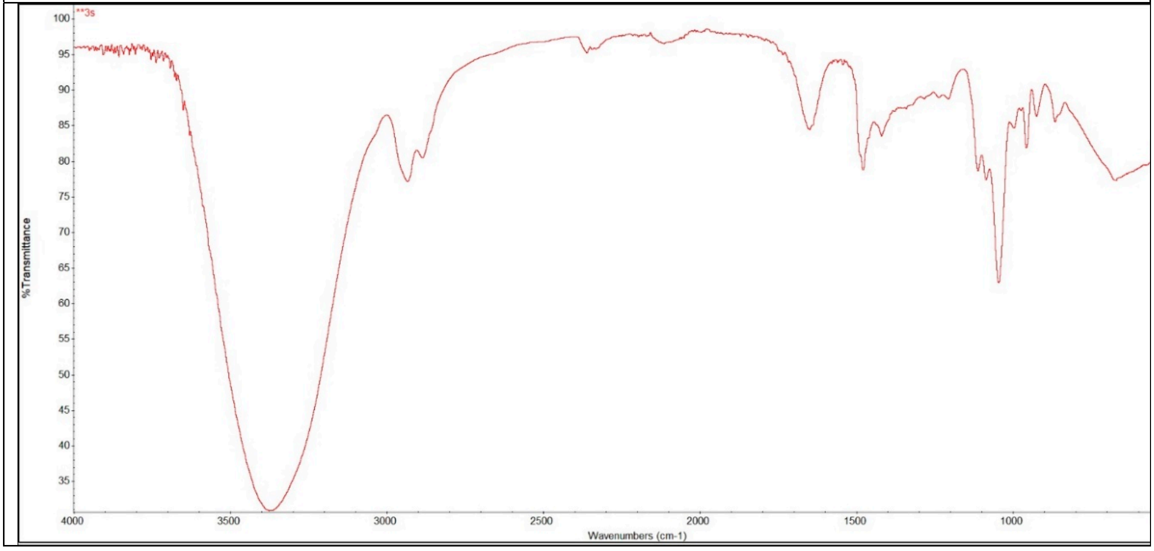

c) ChCl/Glycerol/Water 1:1:2 (NADES 5)

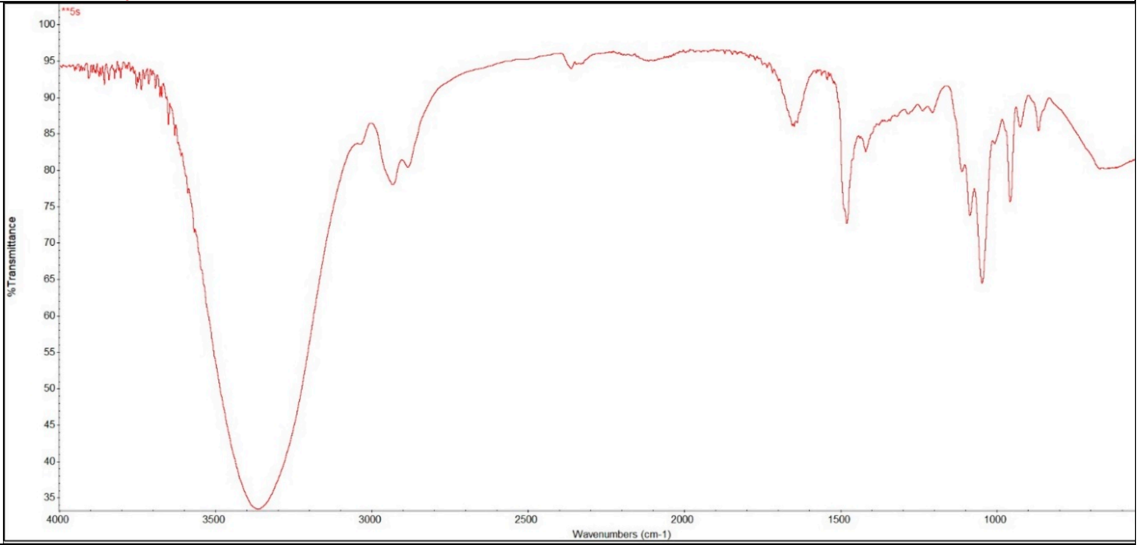

d) ChCl/1,2-propanediol/Water 1:1:1 (NADES 18)

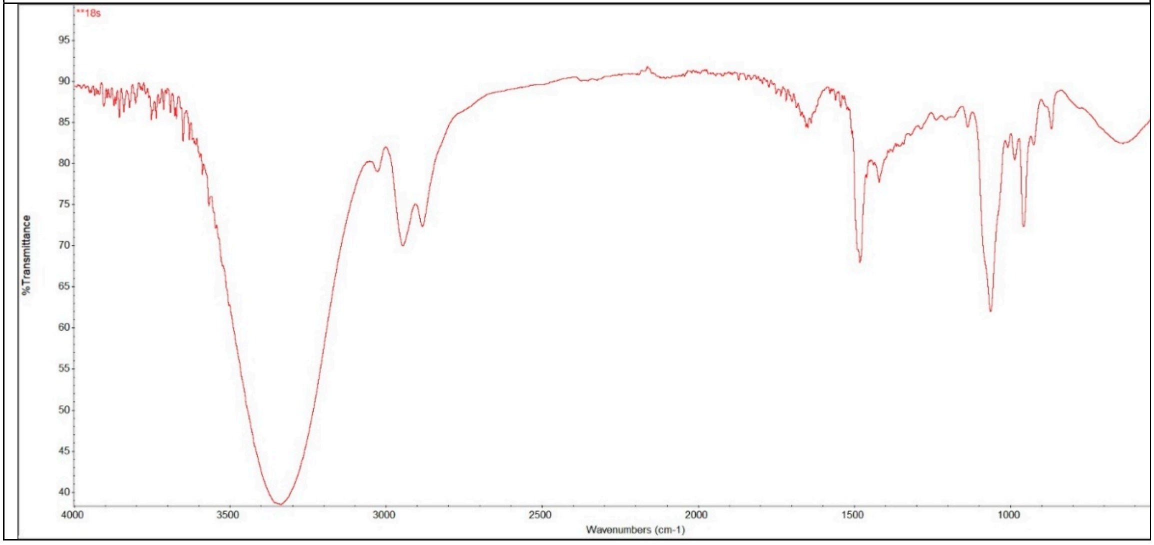

e) ChCl/Glycerol 1:1 (NADES 8)

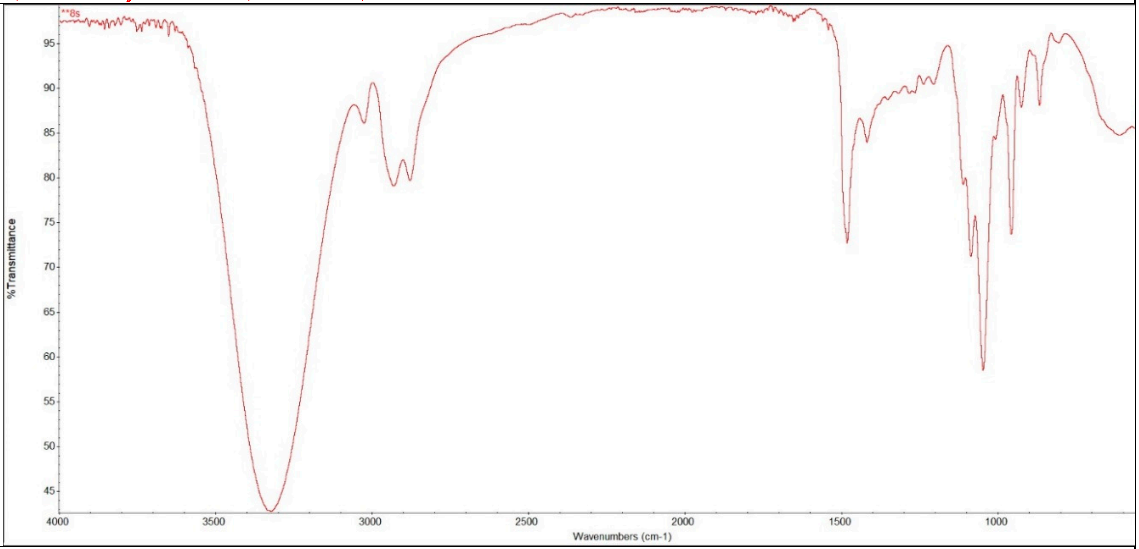

f) ChCl/Glycerol 1:2 (NADES 9)

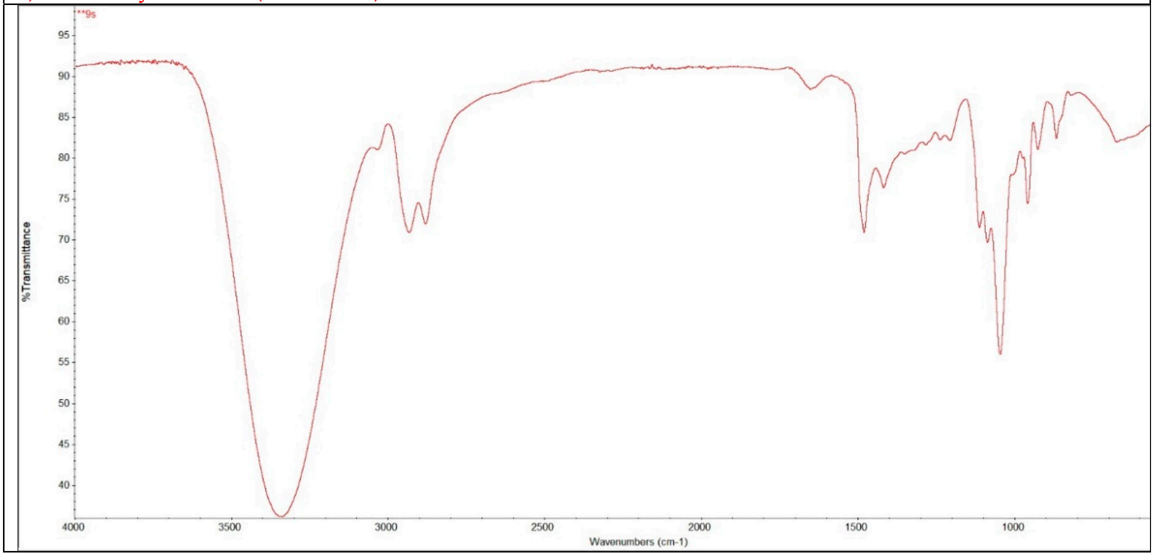

g) ChCl/Glycerol 1:3 (NADES 10)

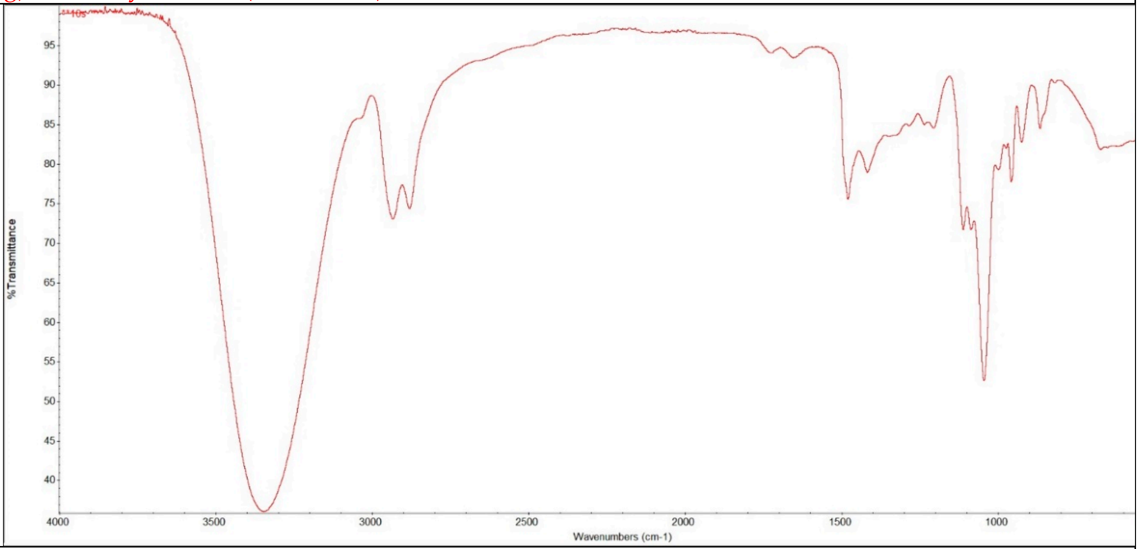

h) ChCl/Fructose/Water 1:1:5 (NADES 2)

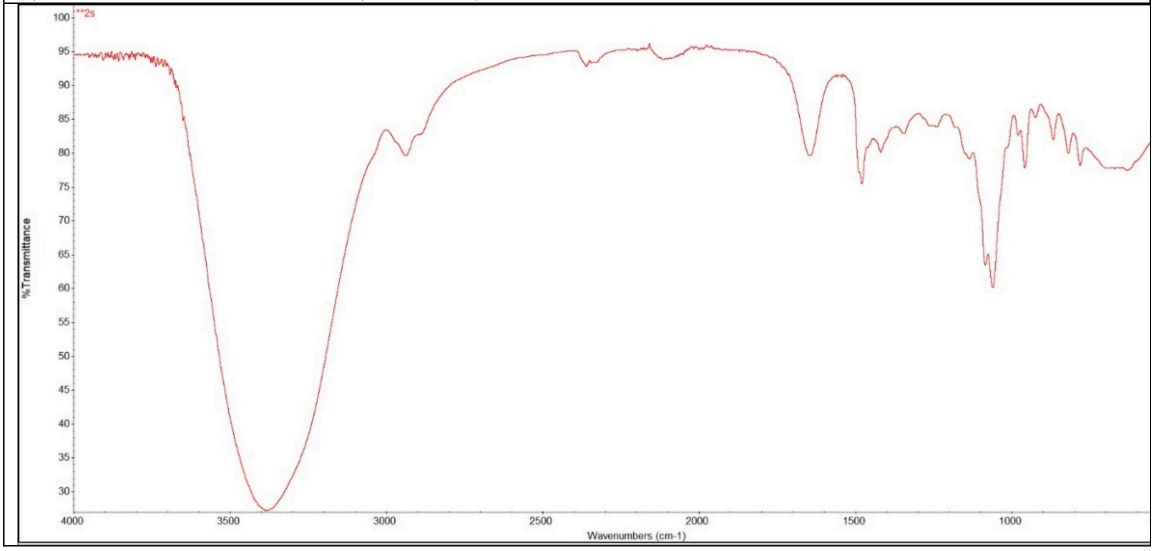

i) ChCl/Glucose/Water 1:1:3 (NADES 4)

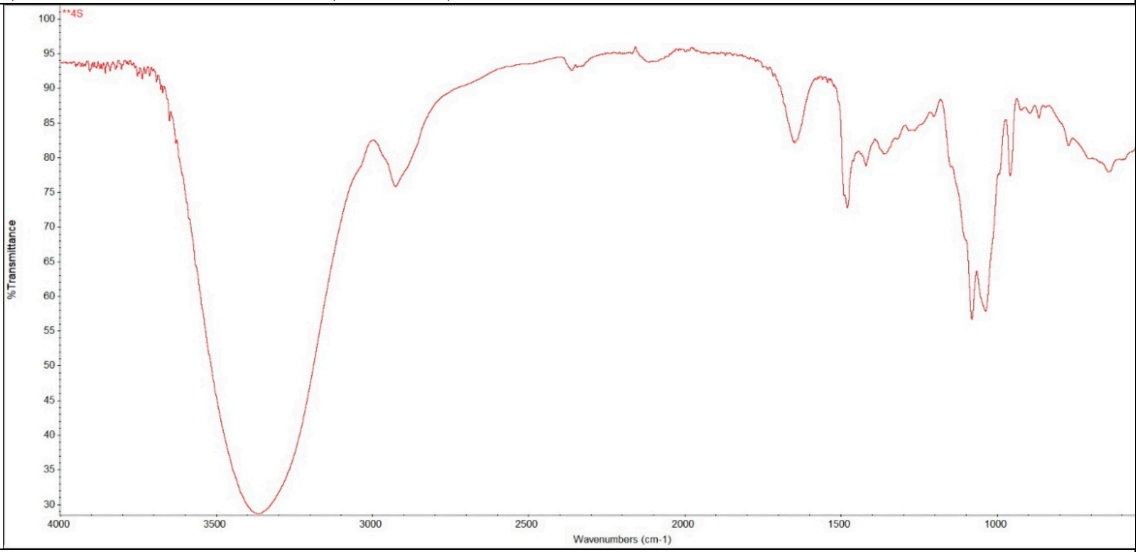

j) ChCl/Xylitol/Water 1:1:5 (NADES 12)

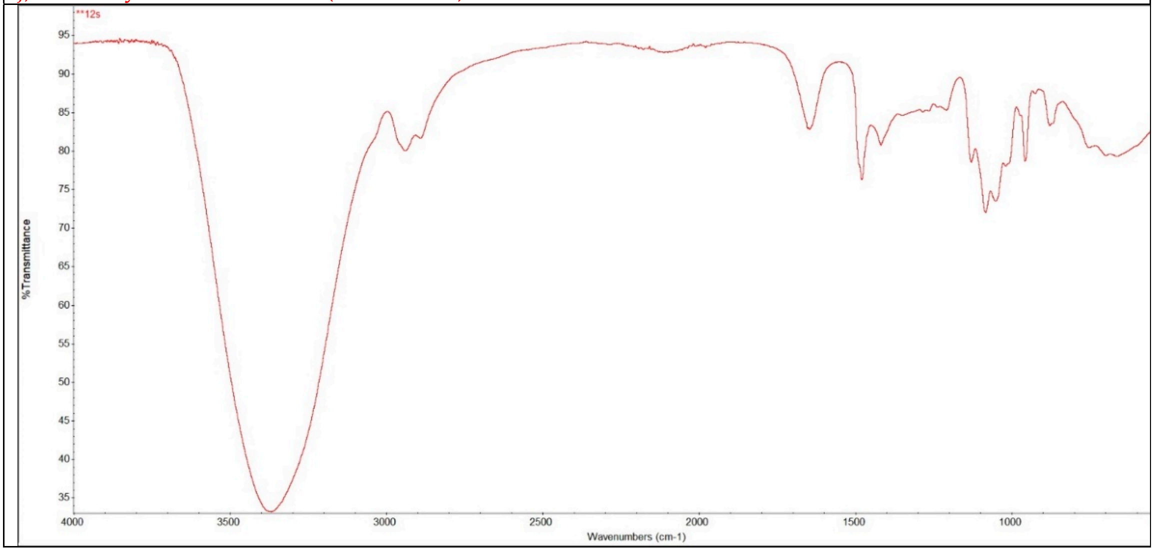

k) ChCl/Lactic acid/Water 1:2:5 (NADES 14)

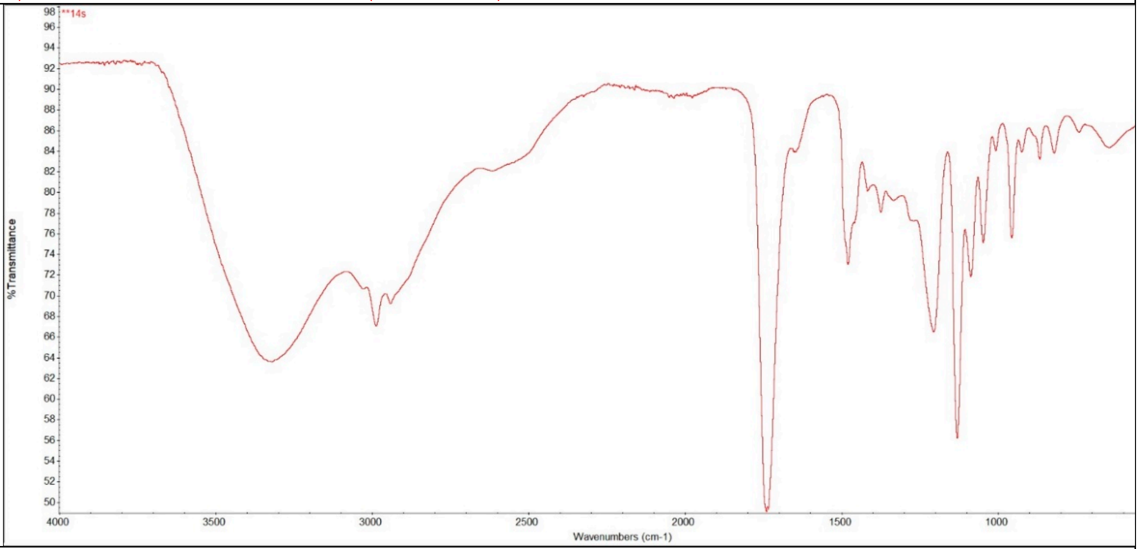

l) ChCl/Citric acid/Water 1:2:5

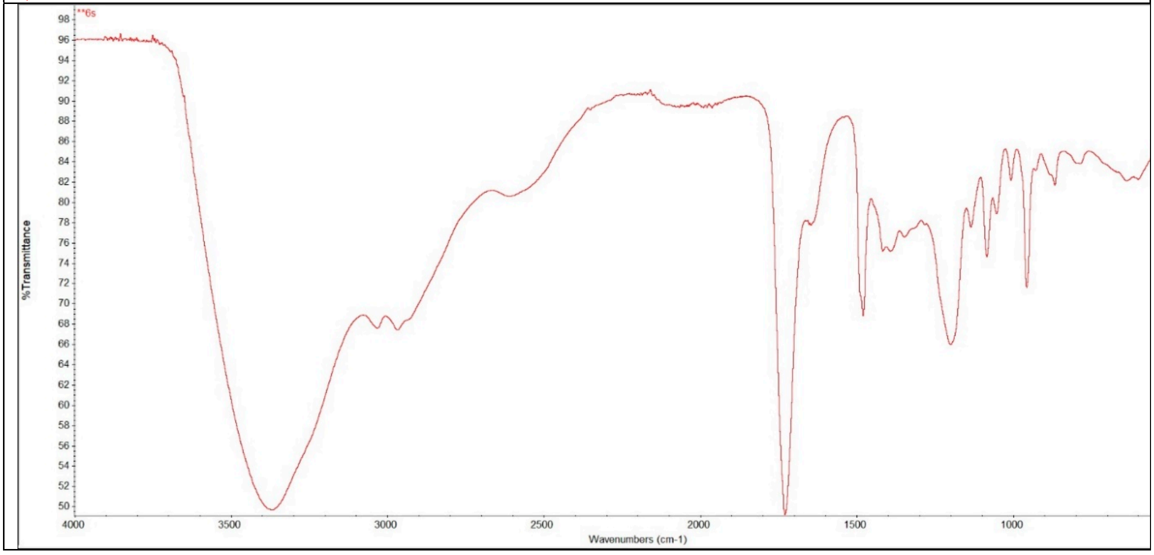

m) ChCl/Citric acid/Water 2:1:5 (NADES 7)

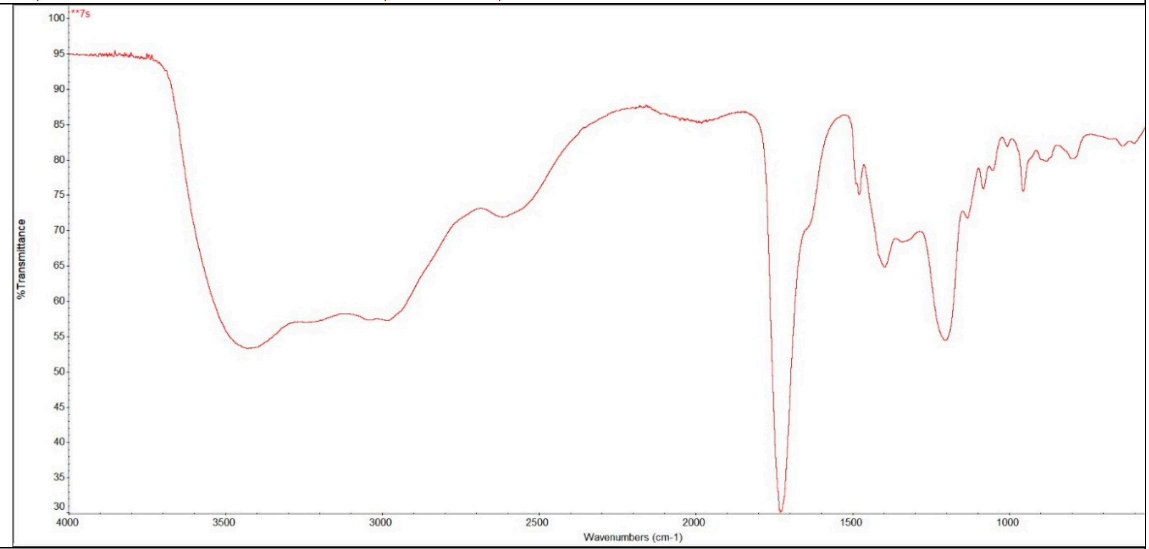

n) ChCl/Citric acid/Water 1:2:3 (NADES 13)

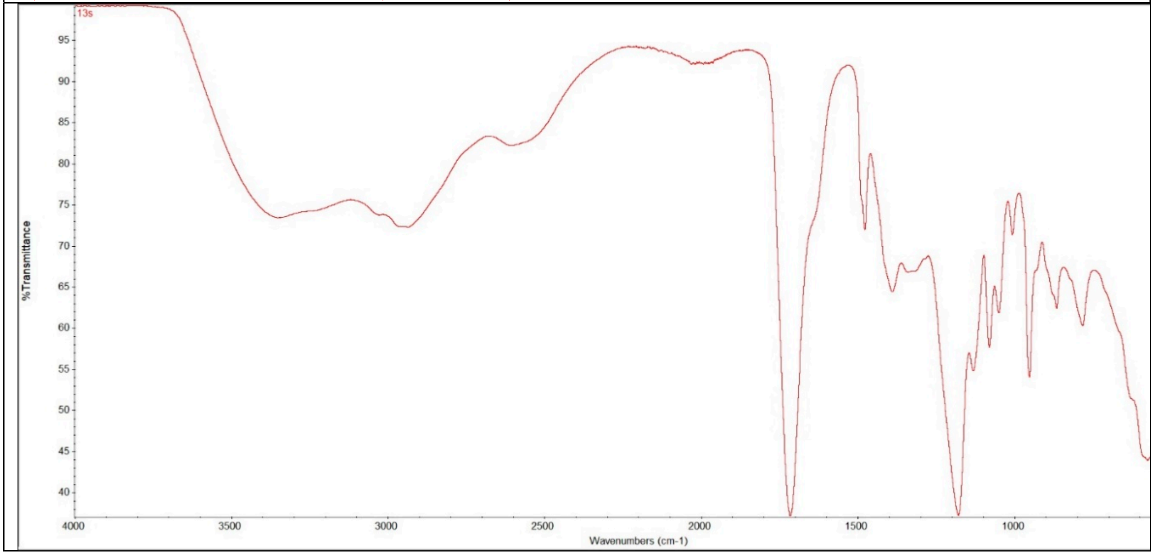

o) ChCl/Citric acid/Water 1:1:5 (NADES 15)

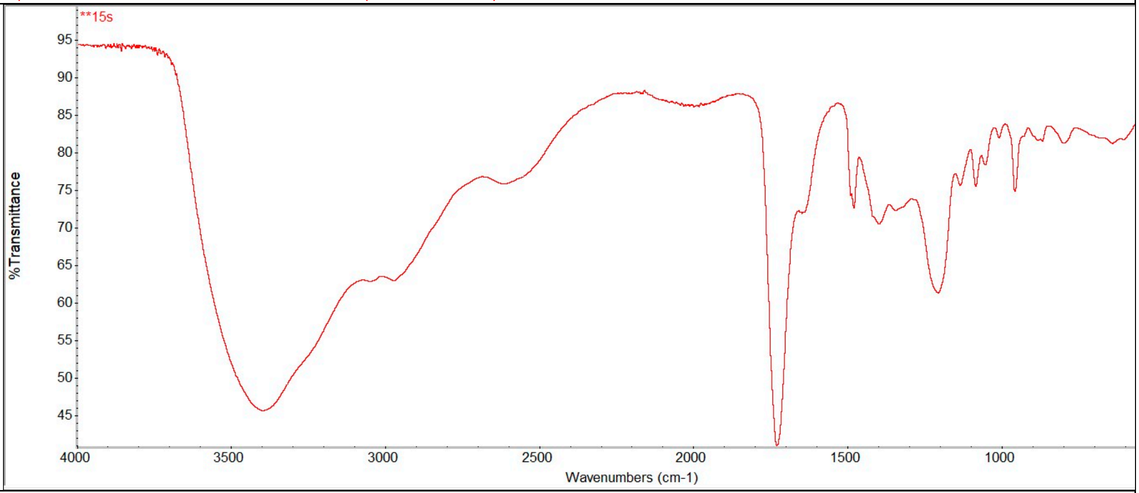

p) ChCl/Glycerol/Citric acid/Water 0.5:2:0.5:5 (NADES 17)

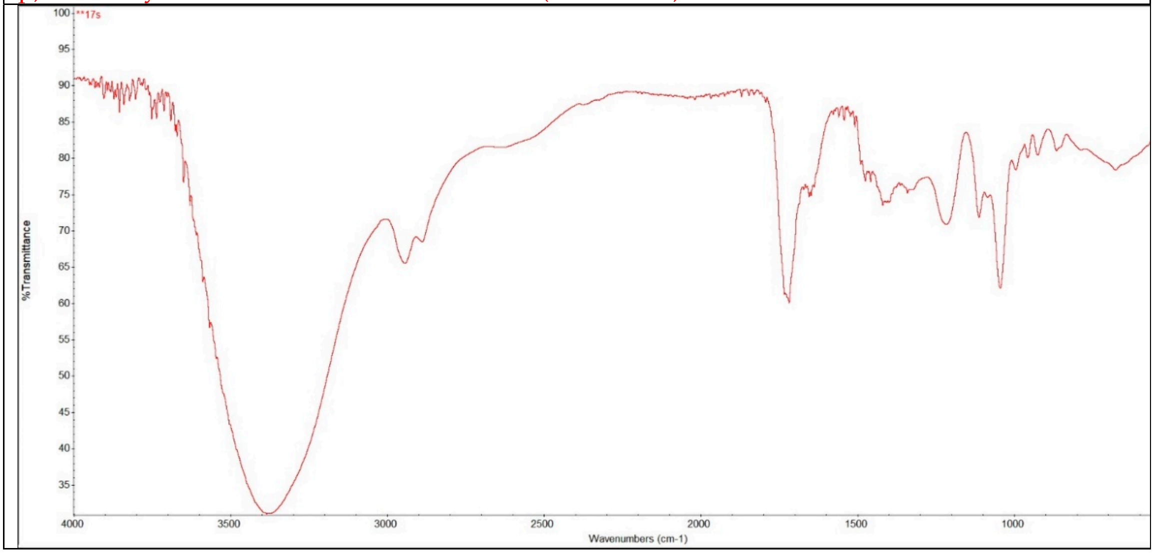

q) ChCl/Urea/Water 1:2:5 (NADES 16)

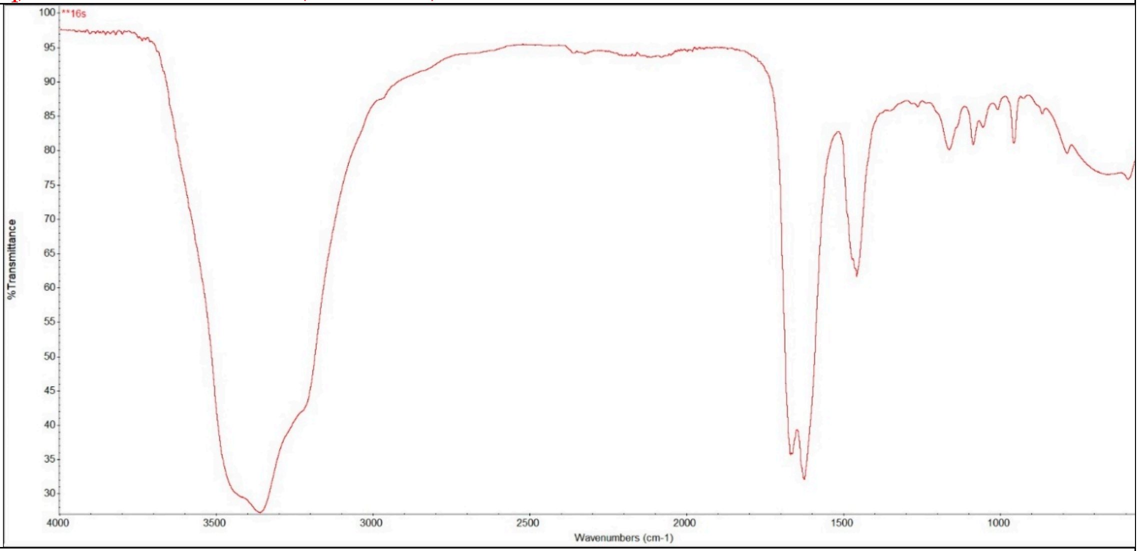

r) Glycerol/Urea/Water 1:1:2 (NADES 11)

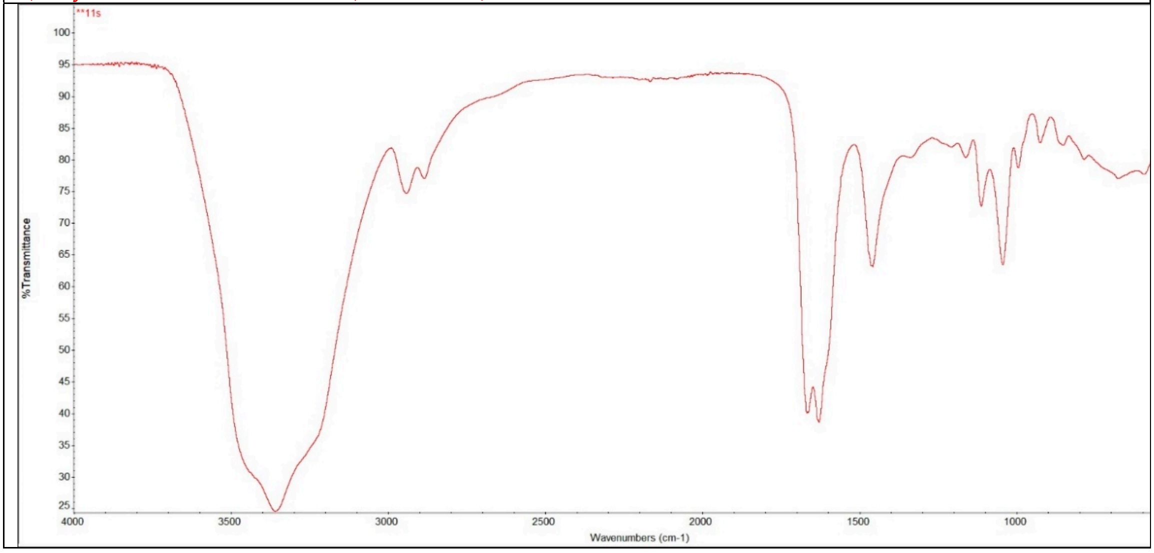

s) Glycerol/Betaine/Water 1:1:3 (NADES 19)

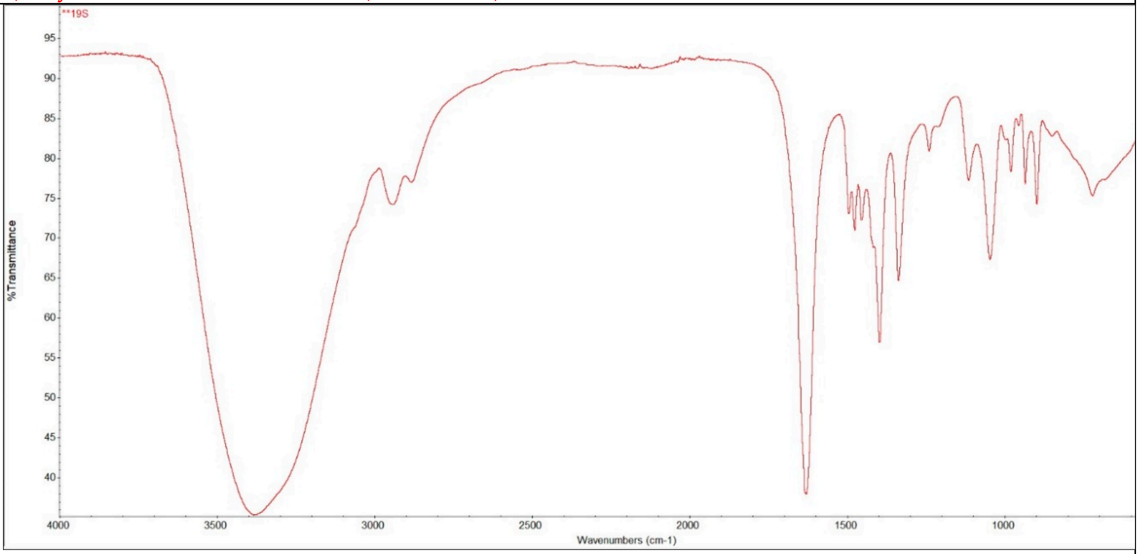

t) Glycerol/Lysine/Water 1:1:3 (NADES 20)

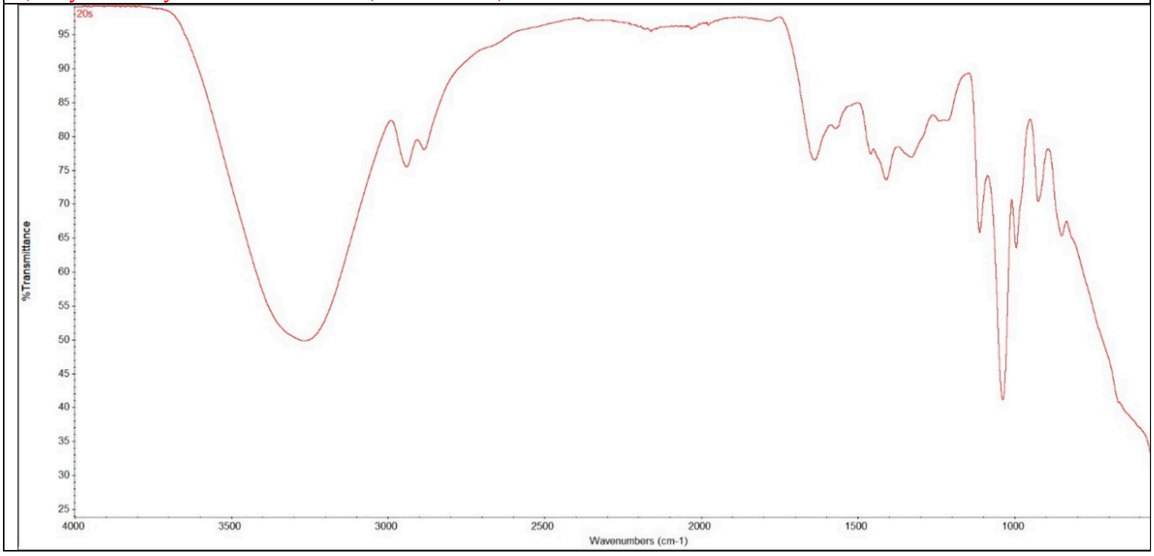

Figure S2. FTIR spectra of NADES mixtures: a) ChCl/Glycerol/Water 1:1:5 (NADES 1); b) ChCl/Glycerol/Water 1:2:5 (NADES 3); c) ChCl/Glycerol/Water 1:1:2 (NADES 5); d) ChCl/1,2-propanediol/Water 1:1:1 (NADES 18); e) ChCl/Glycerol 1:1 (NADES 8); f) ChCl/Glycerol 1:2 (NADES 9); g) ChCl/Glycerol 1:3 (NADES 10); h) ChCl/Fructose/Water 1:1:5 (NADES 2); i) ChCl/Glucose/Water 1:1:3 (NADES 4); j) ChCl/Xylitol/Water 1:1:5 (NADES 12); k) ChCl/Lactic acid/Water 1:2:5 (NADES 14); l) ChCl/Citric acid/Water 1:2:5 (NADES 6); m) ChCl/Citric acid/Water 2:1:5 (NADES 7); n) ChCl/Citric acid/Water 1:2:3 (NADES 13); o) ChCl/Citric acid/Water 1:1:5 (NADES 15); p) ChCl/Glycerol/Citric acid/Water 0.5:2:0.5:5 (NADES 17); q) ChCl/Urea/Water 1:2:5 (NADES 16); r) Glycerol/Urea/Water 1:1:2 (NADES 11); s) Glycerol/Betaine/Water 1:1:3 (NADES 19); t) Glycerol/Lysine/Water 1:1:3 (NADES 20).

Table S1. Percentages of inhibition for tyrosinase and α-amylase. Kojic acid served as the standard for the tyrosinase inhibition assay, while acarbose was used as the reference standard for the α-amylase inhibition assay. Distinct letters next to the corresponding values indicate statistically significant differences ($p < 0.05$) as determined by Tukey's multiple comparisons test.

	NADES composition	Tyrosinase Inhibition (%)	α-Amylase Inhibition (%)
NADES 1	ChCl/Glycerol/Water 1:1:5	22.0 ± 2.7 abg	47.1 ± 0.7 a
NADES 2	ChCl/Fructose/Water 1:1:5	26.1 ± 0.5 b	–
NADES 3	ChCl/Glycerol/Water 1:2:5	13.5 ± 1.1 cge	39.0 ± 0.1 c
NADES 4	ChCl/Glucose/Water 1:1:3	17.4 ± 3.2 ac	–
NADES 5	ChCl/Glycerol/Water 1:1:2	17.4 ± 1.9 ac	36.4 ± 0.1 d
NADES 6	ChCl/Citric acid/Water 1:2:5	96.7 ± 0.2 dh	43.9 ± 0.3 e
NADES 7	ChCl/Citric acid/Water 2:1:5	95.1 ± 0.2 dh	28.5 ± 0.6 f
NADES 8	ChCl/Glycerol 1:1	20.1 ± 8.3 abg	34.7 ± 0.2 g
NADES 9	ChCl/Glycerol 1:2	19.5 ± 1.8 abg	6.4 ± 0.6 h
NADES 10	ChCl/Glycerol 1:3	17.1 ± 3.4 ag	4.7 ± 0.1 i
NADES 11	Glycerol/Urea/Water 1:1:2	8.5 ± 0.5 e	57.3 ± 0.1 j
NADES 12	ChCl/Xylitol/Water 1:1:5	14.0 ± 1.1 age	70.0 ± 0.2 k
NADES 13	ChCl/Citric acid/Water 1:2:3	94.2 ± 1.9 dh	90.7 ± 0.2 l
NADES 14	ChCl/Lactic acid/Water 1:2:5	98.7 ± 0.4 d	90.0 ± 0.2 l
NADES 15	ChCl/Citric acid/Water 1:1:5	89.2 ± 0.3 hi	84.5 ± 0.1 m
NADES 16	ChCl/Urea/Water 1:2:5	21.5 ± 0.5 abg	10.4 ± 0.2 n
NADES 17	ChCl/Glycerol/Citric acid/Water 0.5:2:0.5:5	36.8 ± 3.3 f	36.3 ± 0.1 o
NADES 18	ChCl/1,2-propanediol/Water 1:1:1	26.3 ± 2.9 d	78.6 ± 0.1 p
NADES 19	Glycerol/Betaine/Water 1:1:3	12.0 ± 3.3 ce	35.8 ± 0.1 o
NADES 20	Glycerol/Lysine/Water 1:1:3	38.9 ± 2.9 f	30.8 ± 0.1 q
H_2O		16.9 ± 3.2 ac	27.9 ± 0.1 f
Kojic acid/Acarbose		81.0 ± 2.0 i	86.4 ± 0.1 r

Table S2. Curcumin content expressed as mg/g of dry plant sample ± standard deviation. Distinct letters next to the corresponding values indicate statistically significant differences ($p < 0.05$) as determined by Tukey's multiple comparisons test.

Extract	NADES composition	Curcumin content (mg/g)
NADES 1	ChCl/Glycerol/Water 1:1:5	21.45 ± 1.37 a
NADES 2	ChCl/Fructose/Water 1:1:5	6.65 ± 0.42 b
NADES 3	ChCl/Glycerol/Water 1:2:5	4.90 ± 0.31 bd
NADES 4	ChCl/Glucose/Water 1:1:3	16.00 ± 1.02 ci
NADES 5	ChCl/Glycerol/Water 1:1:2	20.08 ± 1.28 a
NADES 6	ChCl/Citric acid/Water 1:2:5	3.77 ± 0.24 bd

NADES 7	ChCl/Citric acid/Water 2:1:5	5.38 ± 0.34 [bh]
NADES 8	ChCl/Glycerol 1:1	3.95 ± 0.25 [bd]
NADES 9	ChCl/Glycerol 1:2	3.54 ± 0.23 [bd]
NADES 10	ChCl/Glycerol 1:3	3.14 ± 0.20 [bd]
NADES 11	Glycerol/Urea/Water 1:1:2	1.21 ± 0.08 [d]
NADES 12	ChCl/Xylitol/Water 1:1:5	4.82 ± 0.31 [bd]
NADES 13	ChCl/Citric acid/Water 1:2:3	3.65 ± 0.23 [bd]
NADES 14	ChCl/Lactic acid/Water 1:2:5	13.73 ± 0.88 [i]
NADES 15	ChCl/Citric acid/Water 1:1:5	19.95 ± 1.27 [ac]
NADES 16	ChCl/Urea/Water 1:2:5	6.93 ± 0.44 [b]
NADES 17	ChCl/Glycerol/Citric acid/Water 0.5:2:0.5:5	26.53 ± 1.69 [e]
NADES 18	ChCl/1,2-propanediol/Water 1:1:1	30.73 ± 1.96 [f]
NADES 19	Glycerol/Betaine/Water 1:1:3	31.70 ± 2.02 [f]
NADES 20	Glycerol/Lysine/Water 1:1:3	1.30 ± 0.08 [dh]
H_2O		26.91 ± 1.72 [e]
